# Ambiguous Role of Cations in the Long-Term Performance of Electrochemical Capacitors with Aqueous Electrolytes

**DOI:** 10.1021/acsami.2c21926

**Published:** 2023-05-04

**Authors:** Anetta Platek-Mielczarek, Justyna Piwek, Elzbieta Frackowiak, Krzysztof Fic

**Affiliations:** † Institute of Chemistry and Technical Electrochemistry, Poznan University of Technology, Berdychowo 4, 60-965 Poznan, Poland; ‡ Laboratory for Multiphase Thermofluidics and Surface Nanoengineering, Department of Mechanical and Process Engineering, ETH Zurich, Sonneggstrasse 3, 8006 Zurich, Switzerland

**Keywords:** electrochemical capacitor, aqueous electrolyte, sulfate-based capacitors, aging, prolonged lifetime, activated carbon electrode

## Abstract

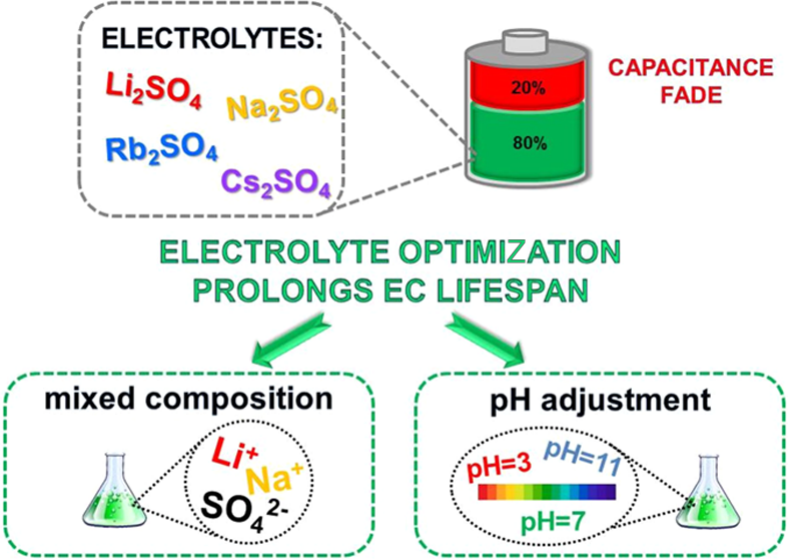

A comprehensive comparison of electrochemical capacitors (ECs) with various aqueous alkali metal sulfate solutions (Li_2_SO_4_, Na_2_SO_4_, Rb_2_SO_4_, and Cs_2_SO_4_) is reported. The EC with a less conductive 1 mol L^–1^ Li_2_SO_4_ solution demonstrates the best long-term performance (214 h floating test) compared to the EC with a highly conductive 1 mol L^–1^ Cs_2_SO_4_ solution (200 h). Both the positive and negative EC electrodes are affected by extensive oxidation and hydrogen electrosorption, respectively, during the aging process, as proven by the *S*_BET_ fade. Interestingly, carbonate formation is observed as a minor cause of aging. Two strategies for optimizing sulfate-based ECs are proposed. In the first approach, Li_2_SO_4_ solutions with the pH adjusted to 3, 7, and 11 are investigated. The sulfate solution alkalization inhibits subsequent redox reactions, and as a result, EC performance is successfully enhanced. The second approach exploits so-called bication electrolytic solutions based on a mixture of Li_2_SO_4_ and Na_2_SO_4_ at an equal concentration. This concept allows the operational time to be significantly prolonged, up to 648 h (+200% compared to 1 mol L^–1^ Li_2_SO_4_). Therefore, two successful pathways for improving sulfate-based ECs are demonstrated.

## 1 Introduction

The high power response and moderate energy output of electrochemical capacitors (ECs) continuously attract scientific attention.^1^ Over the years, many attempts have been made to overcome the limits of their long-term energy production and performance.^2,3^ In this regard, various approaches have been considered, including advancements in porous electrode materials,^4,5^ improvements in electrolyte composition,^6,7^ and electrode/electrolyte matching.^8,9^

Activated carbons (ACs), due to their well-developed surface area, environmental origin (synthesis from raw, natural, abundant precursors), and tunable physicochemical properties,^10^ have been used mainly as electrode materials in ECs. Furthermore, carbon surface can be modified by heteroatom doping^11−13^ or functional group incorporation.^14,15^ Recently, increasing attention has been given to electrode material design and tunability, especially in a sustainable manner. As a consequence, numerous fundamental studies have been conducted, and value-adding insights into processes ongoing at the electrode/electrolyte interface have been provided.^16,17^ Unfortunately, thus far, such findings have not greatly increased the commercial use of ECs. The electric double-layer performance depends on the combination of the active material and electrolytic solution, directly influencing the electrochemical properties of the system (specific capacitance, rate handling, or cycle life). Hence, a detailed understanding of the role of electrolytes in EC performance and aging should be addressed in parallel to material degradation investigations.^18−20^

To promote the sustainability of ECs, aqueous electrolytic solutions have been proposed as an alternative to organic formulations.^21,22^ With water-based electrolytes, the operational voltage of ECs is moderate (ca. 1.2 V); however, the high ionic conductivity of these electrolytes (>50 mS cm^–1^)^11,23,24^ increases the EC power rate. Nevertheless, with pseudocapacitive contributions, the energy density increases but still only approaches the level characteristic of batteries. Moreover, the high sustainability, nontoxicity, and environmental friendliness of aqueous solutions are of great interest, especially compared to their organic counterparts.^18,25^

Water-based ECs have been continuously optimized, and various formulations have been proposed thus far. In particular, when neutral aqueous electrolytes are applied, the corresponding ECs are capable of operating even at a voltage higher than the water decomposition voltage (ca. 1.5–1.8 V) due to the high potentials of hydrogen and oxygen evolution.^26^ It has to be pointed out that to reach such voltage values, the population of ions at the interface is crucial; although the presence of H_3_O^+^ or OH^–^ is essential in terms of formal hydrogen or oxygen evolution potentials, the role of other ions cannot be neglected. To date, mainly nitrate- or sulfate-based systems have been successfully surveyed.^26−28^ They are considered purely capacitive systems, and it has been claimed that positive electrode damage is the main reason for system failure in the long-term perspective.^29−31^ During the operation of the capacitor, the positive electrode is gradually oxidized and eventually corroded.^30^ The specific surface area and electrical conductivity of the electrode decrease as the oxygen content increases (>30%). Furthermore, one of the reasons for system failure (in LiNO_3_- and Li_2_SO_4_-based ECs) was the precipitation of Li_2_CO_3_ from CO_2_ and Li^+^.^31,32^ CO_2_ can be formed in the system from the carbon oxidation/corrosion process as proven by in situ gas spectrometry.^33,34^ Li_2_CO_3_, which is poorly soluble in water, can clog the pores of the electrode and decrease the surface area available for further charge accumulation processes.^35^ For example, inorganic salt deposits were observed on electrodes after long-term floating tests with 1 mol L^–1^ LiNO_3_.^31^

Numerous studies on novel electrode materials in ECs involved the use of lithium or sodium sulfate-based electrolytes as representative aqueous solutions.^36,37^ This experimental approach proves the high reliability of these electrolytes among all aqueous and inorganic electrolytes. Generally, the salt concentration used was 0.5^36^ or 1.0 mol L^–1^.^37−39^ The selection of the cation was determined by investigating sulfate salts with various alkali metal cations (such as Li^+^, Na^+^, and K^+^) and their influence on the electrochemical performance of ECs.^38,40^ Advantageously, not only liquid-state electrolytes but also gel-state electrolytes can be applied in sulfate-based systems.^41^ This approach increases the applicability of aqueous electrolytes in flexible devices, which is expected to become a future research direction of energy storage devices.^42^

However, a comprehensive picture of the aging and discussion of failure mechanisms of capacitors with sulfate-based electrolytes other than 1 mol L^–1^ Li_2_SO_4_ has not yet been reported. Therefore, considering the extensive research conducted on water-based systems,^43^ we have found that several key insights, such as the impact of cations on electrochemical performance (specific capacitance, power ability, or rate handling) and long-term cycling, are still missing.

We attempted to correlate certain cation features with the textural changes detected in the electrodes after aging with EC performance. A novel concept of an electrolyte that consists of an SO_4_^2–^ anion and two cations has also been proposed (called a bication electrolyte). It significantly increased the operating time of ECs and decreased electrochemical changes after reaching the end-of-life criterion (*C*/*C*_0_ = 80%). The results presented in the manuscript complement previous reports on sulfate-based electrolytes in EC applications.^30,32,34,44^ Moreover, we propose two novel approaches for EC lifetime extension.

## 2 Experimental Section

### 2.1 Components of the Electrochemical System

All investigations were performed in Swagelok cells with stainless steel (316 L) current collectors. Microporous carbon cloth KYNOL 507-20, cut into 10 mm diameter disks, was selected as the electrode material, with an average mass of ∼9.5 mg (± 4%). KYNOL 507-20, as a carbon textile, does not require any additives to form a free-standing electrode. For comparison, carbon powder BP2000 was also used as an electrode material, which is fully described in the Supporting Information. Whatman GF/A glass fiber played the role of a separator with a diameter of 12 mm and a thickness of 260 μm. Aqueous solutions of 1 mol L^–1^ sulfate-based salts, i.e., Li_2_SO_4_, Na_2_SO_4_, Rb_2_SO_4_, and Cs_2_SO_4_, were prepared using distilled water. Due to the limited solubility of K_2_SO_4_ (max. concentration: ∼0.5 mol L^–1^), it was not included in the study. All salts were purchased from Sigma-Aldrich, with a purity of >99.8%. Furthermore, 0.5 mol L^–1^ solutions of Li_2_SO_4_ and Na_2_SO_4_ were prepared to study the bication electrolytes with the same concentration of sulfate anion (i.e., 1 mol L^–1^) as in the single-cation electrolytic solutions. Bication mixtures were prepared by mixing appropriate amounts of salts. To prepare 10 mL of the final bication solutions with a concentration of 1 mol L^–1^ Li_2_SO_4_ + 1 mol L^–1^ Na_2_SO_4_, 1.0994 and 1.4204 g of the respective salts were used, while for the 0.5 mol L^–1^ Li_2_SO_4_ + 0.5 mol L^–1^ Na_2_SO_4_ mixture, 0.5497 and 0.7102 g of the respective salts were used. The mixed salt powders were then dissolved using distilled water in a volumetric flask (10 mL). To prepare the final electrolytic bication solutions, two mixtures were used:

(1) Mixture 1: Li^+^ = 1 mol L^–1^, Na^+^ = 1 mol L^–1^, SO_4_^2–^ = 2 mol L^–1^.
(2) Mixture 2: Li^+^ = 0.5 mol L^–1^, Na^+^ = 0.5 mol L^–1^, SO_4_^2–^ = 1 mol L^–1^.

For clarity, mixture 1 and mixture 2 are used in the following.

### 2.2 Testing Protocol

The EC performance was compared based on cyclic voltammetry results (1–100 mV s^–1^), constant-current charge/discharge profiles (0.1–10 A g^–1^) in the voltage range of 0–0.8 V, and impedance spectroscopy results (recorded at 0 V in the frequency range of 100 kHz to 1 mHz with an amplitude of ±5 mV). Subsequently, the systems were subjected to a constant polarization hold protocol (floating) at an elevated voltage of 1.6 V, which is the main scientific investigation in this manuscript. This voltage was selected based on data reported in the literature.^32,33,38^ Furthermore, stepwise voltage window extension (by +0.1 V from 0.8 to 2.0 V) was carried out using cyclic voltammetry (2 mV s^–1^) and constant-current charge/discharge (0.1 A g^–1^). The aging protocol consisted of performing cyclic voltammetry at a scan rate of 5 mV s^–1^ (0–0.8 and 0–1.6 V), constant-current charge/discharge at 1 A g^–1^ (0–1.6 V), and impedance spectroscopy at 0 V, controlled every 2 h of floating as long as the system met its end-of-life criterion. Such a procedure allowed for the analysis of a full aging process (quantitative and qualitative). The end-of-life criterion was defined as a 20% decrease in specific capacitance, as suggested by the International Standard.^45^

Gas chromatography–mass spectrometry (GC–MS) analyses were conducted using an EVOQ GC-triple quadrupole (GC-TQ) MS, Bruker. The apparatus was connected with a PAT-Cell-Gas made by EL-CELL. The temperature of the injector was 175 °C, that of the column was 150 °C, and that of the mass spectrometer was 220 °C. The electrochemical cell was constructed of two binder-free electrodes (KYNOL 507-20) with a diameter of 16 mm separated by a glass fiber separator (Whatman GF/A, thickness: 260 μm). The amount of electrolyte used was 250 μL. The electrochemical cell was connected to the GC–MS apparatus to monitor the gas evolution in operando mode. Cyclic voltammetry was carried out in the voltage range of −1.6 to +1.6 V at a scan rate of 1 mV s^–1^.

### 2.3 Physicochemical Characterization

Electrolytic solutions were characterized in terms of their pH and conductivity using a pH meter and Mettler Toledo SevenCompact conductometer. The electrode material was characterized in terms of its textural properties by N_2_ adsorption/desorption at 77 K. Prior to textural analysis after aging, the EC systems were dismounted, the electrodes were separated, and the electrodes were washed extensively with distilled water at room temperature. After this step, the electrodes were predried to remove excess moisture. The samples were then degassed initially under He flow at 100 °C for 24 h and subsequently under vacuum for 5 h before recording the adsorption isotherm. The specific surface area, *S*_BET_, was calculated using the Brunauer–Emmett–Teller equation in the pressure range of 0.01–0.05. For this purpose, the ASAP 2460 apparatus with Micromeritics software was used. The pore size distribution and cumulative surface area, *S*_CUM_, were calculated using the SAIEUS program (version 2.0, Micromeritics, 2D non-local density functional theory (2D-NLDFT) model). The average pore diameter (*L*_0_) was determined with the maximum peak method. Appropriate error bars are included in the plots related to the textural characteristics of the material.

## 3 Results and Discussion

### 3.1 Alkali Metal Sulfate Electrolytes for EC Application

Initially, the influence of alkali metal cations on the electrochemical behavior of ECs is elucidated using 1 mol L^–1^ solutions of M_2_SO_4_ (M = Li^+^, Na^+^, Rb^+^, Cs^+^). The salt properties and physicochemical characterization of these aqueous solutions are summarized in Table 1.

**Table 1:** Physical and Chemical Properties of Various Cations and Aqueous Solutions of Alkali Metal Sulfate Salts (1 mol L^–1^)^46−51^

	Li_2_SO_4_	Na_2_SO_4_	Rb_2_SO_4_	Cs_2_SO_4_
molar mass, g mol^–1^	110	142	267	362
solubility, g/100 mL H_2_O	35	28	51	167
maximum concentration, mol L^–1^	2.3	1.5	1.2	1.7
	1 mol L^–1^ aqueous solution			
pH	8	6	4	10
conductivity, mS cm^–1^	72	83	150	150
	Li^+^	Na^+^	Rb^+^	Cs^+^
solvated cation radius, nm	0.382	0.358	0.329	0.329
cation hydration enthalpy, kJ mol^–1^	520	406	520	276
cation binding energy to carbon aromatic ring, kcal mol^–1^	38	28	38	16

**Figure 1:**
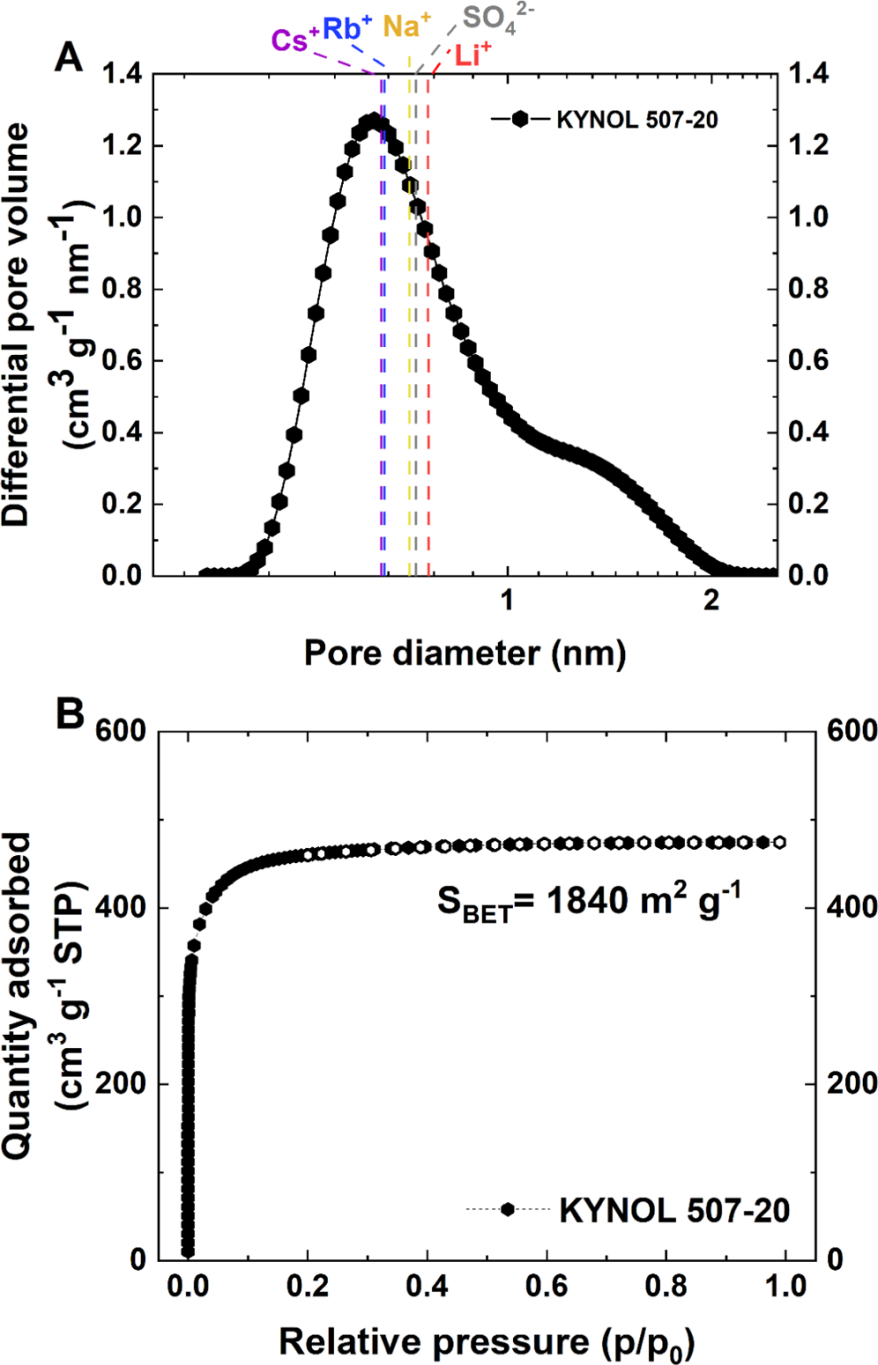
Textural characterization of KYNOL 507-20: (A) pore size distribution calculated from N_2_ adsorption at 77 K with presumed ion dimensions present in the electrolytic solution; (B) adsorption isotherms with the BET specific surface area value.

Alkali metal sulfate solutions are easily soluble in water. The diameter of the solvated cation increases in the following order: Li^+^ > Na^+^ > Rb^+^ = Cs^+^. However, knowing the textural properties of the pristine carbon cloth (*S*_BET_ = 1840 m^2^ g^–1^ and *V*_MICRO_ = 0.70 cm^3^ g^–1^; Figure 1), the composition of the electric double-layer (EDL) at the electrode/electrolyte interface can be assumed (calculations presented in Table S1).

The hydration enthalpy of cations shows that the larger the cation dimension is, the easier the cation is desolvated; larger cations (such as Cs^+^) are more prone to losing their solvation shell than smaller cations (such as Li^+^).

Furthermore, for alkali metals in the order from Li^+^ to Cs^+^, one may notice that cation–carbon π bonding interactions become weaker. Thus, Li^+^ can be adsorbed on the carbon surface more strongly than Cs^+^. This might induce structural changes in carbon during long-term charging/discharging or affect the efficiency of the charging/discharging process. Therefore, cation–carbon π bonding interactions are discussed considering the energetic efficiency of charging/discharging at various voltages (0.8 and 1.6 V) and current densities for the 1 mol L^–1^ Li_2_SO_4_ and Cs_2_SO_4_ electrolytes.

Furthermore, regarding the ionic conductivities of the 1 mol L^–1^ solutions, the Li_2_SO_4_ and Na_2_SO_4_ solutions demonstrate rather moderate values of 72 and 83 mS cm^–1^, while both the Rb_2_SO_4_ and Cs_2_SO_4_ solutions demonstrate remarkably higher values, i.e., ∼150 mS cm^–1^.

On the one hand, a 1 mol L^–1^ aqueous solution of Cs_2_SO_4_ can be expected to provide the highest capacitance value (excess charge carriers in the electrolyte volume, high conductivity value). On the other hand, relatively large ions can cause adsorption failure when penetrating small ultramicropores. Thus, a detailed textural characterization of carbon electrodes is of high importance.

The long-term performance of sulfate-based ECs has been verified for systems with KYNOL 507–20 carbon cloth as electrodes. Figure 1A presents the pore size distribution of KYNOL 507-20, together with the assumed solvated cation diameters in the micropore region.

The sulfate anion, with a diameter of ∼0.76 nm,^50^ is the largest species in the electrolytic solution. However, all ions in the solvated state can penetrate the pores of KYNOL 507-20.

The nitrogen adsorption isotherm at 77 K is presented in Figure 1B. The value shown is the *S*_BET_ value of 1840 m^2^ g^–1^ for KYNOL 507-20. It is worth mentioning that the *S*_CUM_ of this material is 1635 m^2^ g^–1^. Carbon cloth is purely microporous with a type I isotherm (IUPAC classification),^52^ a narrow pore size distribution up to 2 nm, an average micropore diameter *L*_0_ equal to 0.75 nm, and a high micropore volume of 0.70 cm^3^ g^–1^.

**Figure 2:**
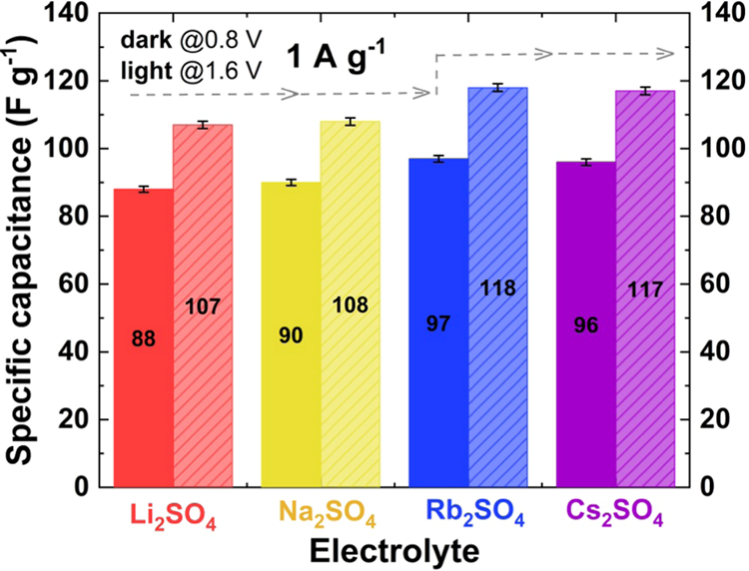
Specific capacitance calculated at 0.8 (dark bar) and 1.6 V (light bar) at 1 A g^–1^ for ECs with 1 mol L^–1^ M_2_SO_4_ (where M = Li^+^, Na^+^, Rb^+^, or Cs^+^) and KYNOL 507-20 electrodes.

The galvanostatic charge/discharge test results are summarized in Figure 2. It is assumed that all observed phenomena are related only to the interactions of carbon/electrolyte ionic species since KYNOL 507-20 is a self-standing carbon cloth. ECs with such carbon and sulfate-based electrolytes show good rate handling, satisfactory specific capacitance, and balanced potential distribution between electrodes.

The specific capacitance calculated at 0.8 V slightly increases from the value of 88 F g^–1^ recorded for Li_2_SO_4_ to 96 F g^–1^ recorded for Cs_2_SO_4_, as presented in Figure 2. A higher specific capacitance is observed when Cs_2_SO_4_ solution is used as an electrolyte, which originates from the combined effect of electrolytic properties and selected carbon texture.^46^ The same trend is observed for the specific capacitance in the wide voltage range of 0–1.6 V. Furthermore, two levels of specific capacitance can be observed for the data recorded at 0.8 and 1.6 V. For ECs with 1 mol L^–1^ Li_2_SO_4_, a specific capacitance similar to that of the EC with 1 mol L^–1^ Na_2_SO_4_ is observed. These two electrolytes are characterized by similar ionic conductivities (72 vs 83 mS cm^–1^) and solvated cation dimensions. The ECs operating with electrolytic solutions of Cs_2_SO_4_ and Rb_2_SO_4_ are also characterized by similar specific capacitances at both voltages. The size of the cation present in the electrolytic solution and its affinity for carbon π bonding can be crucial. From this point of view, Cs^+^ seems to be weakly attracted to carbon, and its desolvation process proceeds more easily than that of Li^+^, so its neat diameter might be even smaller than that of the solvated lithium cation. However, the specific capacitances of the ECs with 1 mol L^–1^ Rb_2_SO_4_ and Cs_2_SO_4_ both surpass that of the EC with 1 mol L^–1^ Li_2_SO_4_. For these aqueous solutions, their ionic conductivities are similar (150 mS cm^–1^). Therefore, when microporous carbon plays the role of the electrode material, the main factor that influences the specific capacitance is the conductivity of the applied electrolyte when the ions are small enough to penetrate the carbon micropores. Moreover, the charge/discharge coulombic efficiency, calculated from different electrochemical techniques, seems to be almost the same (95%) for all studied electrolytes. For each sulfate-based system studied, the energetic efficiency during charging/discharging up to 1.6 V at 1 A g^–1^ is as follows: 88% (Li_2_SO_4_), 88% (Na_2_SO_4_), 83% (Rb_2_SO_4_), and 87% (Cs_2_SO_4_). Taking into account these similar values and cation–carbon π bonding energies (Table 1), it can be assumed that the bonding energy does not remarkably affect the energetic efficiency of the charging/discharging process.

After the initial assessment, all sulfate-based ECs were subjected to an accelerated aging protocol (floating). The relative specific capacitance vs floating time of each sulfate-based system is presented in Figure S4. It can be observed that ECs with Na_2_SO_4_ solution operate for the shortest time (116 h), whereas the others operate for much longer, i.e., 180 h (Rb_2_SO_4_), 200 h (Cs_2_SO_4_) and 216 h (Li_2_SO_4_). One of the possible explanations for the capacitor failure with sulfate-based electrolytes is the formation of carbonates,^32,34^ similar to nitrate-based ECs,^31,33^ due to the evolution of CO and CO_2_ in the cell operating at high voltages. Therefore, as water decomposition is presumed to be one of the major factors contributing to the aging process, the formation of carbonate salts in the studied sulfate-based systems is considered (Table 2). The precipitation of carbonate deposits (e.g., Li_2_CO_3_) results in pore clogging that leads to a decrease in accessible surface area for further ion adsorption.^31^

**Table 2:** Physicochemical Properties of Various Alkali Metal Carbonates

	Li_2_CO_3_	Na_2_CO_3_	Rb_2_CO_3_	Cs_2_CO_3_
molar mass, g mol^–1^	74	106	231	326
solubility, g/100 mL H_2_O	1.0	30.7	450.0	260.5
maximum concentration, mol L^–1^	0.1	2.2	3.5	2.2

It seems that only the formation of Li_2_CO_3_ can be responsible for the EC system fade due to its very low solubility value. Therefore, even a small amount of carbon dioxide produced during electrochemical operation in 1 mol L^–1^ Li_2_SO_4_ may lead to system deterioration, as has already been reported.^29,34^ The other salts, i.e., Na_2_CO_3_, Rb_2_CO_3_, or Cs_2_CO_3_, are characterized by a relatively high solubility in water, allowing them to generate solutions with concentrations exceeding 2 mol L^–1^. Thus, even if they are produced during system operation, they instantaneously become dissolved in the electrolyte, influencing only the chemical composition of the electrolyte. Although Li_2_CO_3_ formation has already been proven to be the reason for the failure of ECs with Li_2_SO_4_ electrolyte,^29,34^ this type of system exhibits the longest operating time among other systems based on 1 mol L^–1^ sulfate solutions. Given that each sulfate-based electrolyte undergoes different aging processes as carbonate precipitation is not an obvious reason for aging at 1.6 V.

**Figure 3:**
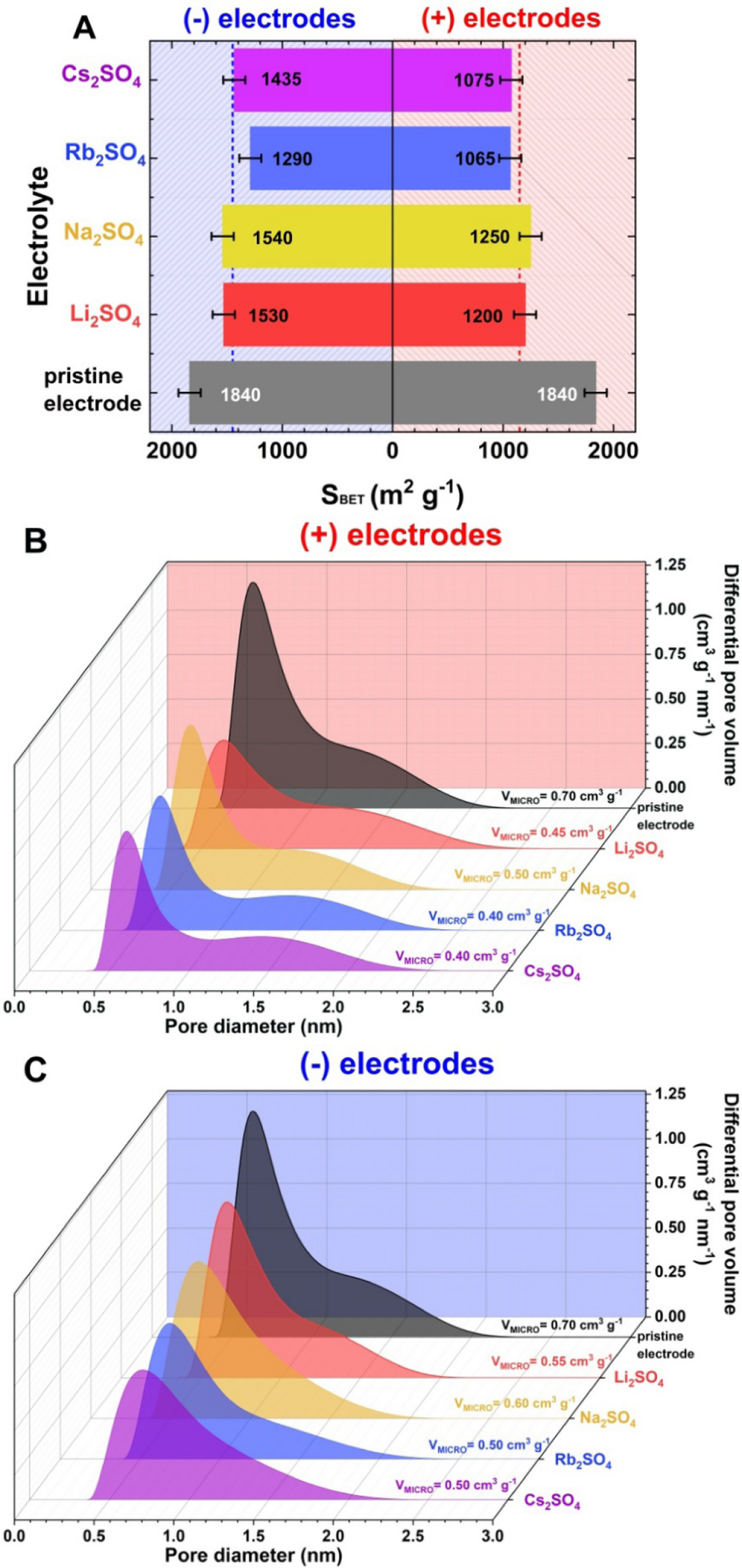
Textural characterization of (−) and (+) electrodes after the aging process of ECs with various sulfate-based electrolytes: (A) specific surface area (*S*_BET_); (B) pore size distribution of (+) electrodes; (C) pore size distribution of (−) electrodes.

To verify these findings, postmortem analysis of the electrode surface area is implemented. It is expected that with no pore clogging by the carbonate precipitate, the *S*_BET_ should decrease only negligibly for negative electrodes while significantly changing for positive electrodes. Figure 3A summarizes the specific surface area of negative (−) and positive (+) electrodes after aging tests. In the case of the nitrate-based system, only the positive electrode is responsible for aging phenomena, and moreover, lithium carbonate precipitation is evidenced in scanning electron microscopy (SEM) micrographs.^31^ However, sulfate-based systems operate with a completely different energy storage mechanism than nitrate-based systems, such as:

(1) No carbonate precipitation is predicted to be found when 1.6 V is applied for systems with Na^+^, Rb^+^, and Cs^+^ cations.
(2) Both electrodes are affected by the aging process (presented in Figure 3).

Positive electrodes are more affected by the aging process, as their overall *S*_BET_ loss is approx. 37.5% (the average *S*_BET_ of the positive electrodes in all sulfate-based systems is equal to 1150 m^2^ g^–1^ and is denoted with a red dashed line shown in Figure 3A). The *S*_BET_ of the negative electrodes equals 1449 m^2^ g^–1^ (Figure 3A) and is smaller than that of the positive electrodes (21%). In summary, positive electrodes age more intensely; however, the deterioration of textural properties is also observed for negative electrodes.

Li^+^ is supposed to form the strongest bond to the carbon network. However, the *S*_BET_ of the negative electrode operating with 1 mol L^–1^ Li_2_SO_4_ is almost the same as that operating with 1 mol L^–1^ Na_2_SO_4_, and both present the highest values recorded for negative electrodes after EC aging. Thus, even Li^+^ is strongly attracted to carbon, its size is small enough to allow easy cation repulsion when the discharge process occurs. The pore size distribution of the aged electrodes is presented in Figure 3B,C. The micropore volume of the negative electrode (Figure 3C) decreases slightly from 0.7 cm^3^ g^–1^ (pristine electrode) to 0.55 cm^3^ g^–1^ (Li_2_SO_4_) and 0.60 cm^3^ g^–1^ (Na_2_SO_4_). No ions are trapped in the pores, and a moderate change in S_BET_ is detected after the aging process. A significant decrease in the *S*_BET_ of negative electrodes operating in Rb_2_SO_4_ (1290 m^2^ g^–1^) and Cs_2_SO_4_ (1435 m^2^ g^–1^) proves that these ions are too large to reversibly penetrate the micropores of carbon cloth or are strongly confined inside. The micropore volume decreases to 0.50 cm^3^ g^–1^ for systems based on Rb_2_SO_4_ and Cs_2_SO_4_, changing the pore size distribution of the aged electrodes. Interestingly, small mesopores (close to 2 nm) are only observed for these two systems (0.06 cm^3^ g^–1^), proving the movement of cations in the pores. This is a result of Cs^+^ and Rb^+^ adsorption in the electrode pores, owing to their size and probable confinement in the pores. Presumably, pore clogging results from potential-driven ion confinement in the textured carbon, as carbonate deposits cannot form in these electrolytes. Textural changes from hydrogen electrosorption could also be considered.

For positive electrodes after aging in 1 mol L^–1^ Li_2_SO_4_ (Figure 3B), the micropore volume is equal to 0.45 cm^3^ g^–1^, while in Na_2_SO_4_, it is 0.50 cm^3^ g^–1^. The electrodes operating in sulfate solutions with Rb^+^ and Cs^+^ cations show a micropore volume of 0.40 cm^3^ g^–1^. It is noteworthy that for the charging of positive electrodes, not only SO_4_^2–^ but also OH^–^ ions should be considered. Various in situ techniques have already confirmed that the real size of OH^–^ as well as that of solvated cations and anions is strongly affected by the electrolyte composition.^44,53,54^ Therefore, it can be predicted that for aqueous solutions of Rb_2_SO_4_ and Cs_2_SO_4_, OH^–^ is surrounded by more water molecules. Interestingly, SO_4_^2–^ ionic flux has not been observed in Li_2_SO_4_ electrolyte,^32^ and according to studies conducted with KI-based electrolyte,^53^ it can be assumed that the charge storage mechanism is the same when considering other alkali metal sulfates.

The performance of the EC electrode with two carbons and a Cs_2_SO_4_ solution is shown in Figures S2 and S3. Faradaic contribution is observed for the positive electrode in the region of 0.4 V vs normal hydrogen electrode (NHE). It correlates with the fade of positive electrode textural properties, which is more pronounced than that of the negative one.

Summarizing the textural characterization of aged electrodes, one can conclude that the aging mechanism does not originate from carbonate formation only, as the time of operation for various sulfate-based ECs at 1.6 V contradicts this hypothesis. ECs with Li_2_SO_4_ and Cs_2_SO_4_ operate for almost the same time; however, the solubility of Li_2_CO_3_ is much lower than that of Cs_2_CO_3_. Thus, most likely, Li_2_CO_3_ is not formed when a voltage of 1.6 V is applied. Moreover, a decrease in *S*_BET_ was observed for both positive and negative electrodes. Such a phenomenon is initially assumed to be observed for only positive electrodes because they are directly subjected to oxidizing conditions. In the effect of the oxidation process, the surface chemistry of the positive electrodes is likely to change, limiting the accessible surface area for further ion adsorption. For the negative electrodes, where most cations are adsorbed/desorbed during electrochemical operation, a decrease in the *S*_BET_ can be explained by cation confinement in the pore volume and possible anion-specific adsorption onto the electrode surface.

**Figure 4:**
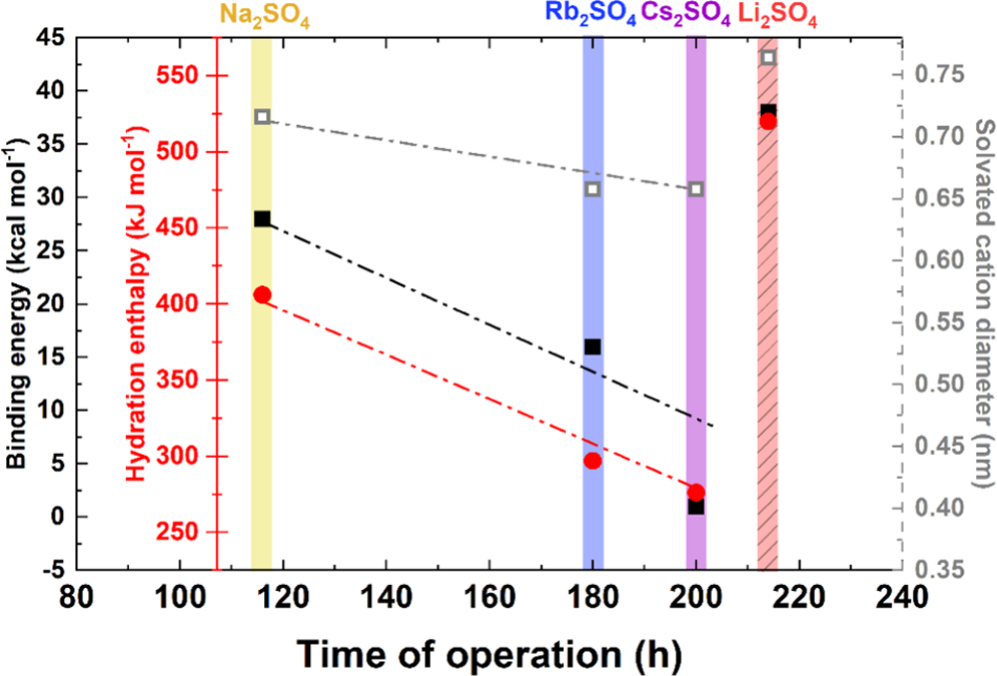
Correlation of various cation features vs operation time of ECs with 1 mol L^–1^ M_2_SO_4_ aqueous solution (M = Li^+^, Na^+^, Rb^+^, and Cs^+^).

A graphic representation of various cation characteristics with EC operation time is shown in Figure 4. The cation parameters considered in Figure 4 are the binding energy to the carbon π bond, the hydration enthalpy, and the diameter of the solvated cation (Table 1).

Considering the binding energy (black squares), it can be observed that the order of Na^+^ > Rb^+^ > Cs^+^ is somehow related to their position in the periodic table. A smaller binding energy means a longer operational time. Thus, ions are more easily adsorbed/desorbed from the electrode/electrolyte interface, and in effect, the ECs operate longer. This can influence the cation confinement in the pore volume, especially because a high binding energy can decrease the charge/discharge efficiency.

Ions characterized by a lower hydration enthalpy tend to be in a desolvated state, and their effective diameter can be smaller (Na^+^ > Rb^+^ > Cs^+^) as a result of different solvation states. It seems that fully desolvated Cs^+^ is smaller than partially desolvated Na^+^, easily penetrating the textured carbon electrodes. However, this observation seems to be reasonable only when discussing the EC time of operation. From the *S*_BET_ change, it appears that Cs^+^ is more likely to be trapped in the pores, causing the clogging of pore volume. Therefore, two contrary explanations of aging are possible. Either Cs^+^ ions are strongly confined in the electrode micropores because of spatial hindrance, or they are easily adsorbed/desorbed because of the high desolvation probability and small effective size of cations. Electrochemical operation cannot be simplified to exact charge separation (as the carbon surface is not neutral and contains functional groups). Moreover, ions are mixed, and the perm-selectivity failure of carbon electrodes is widely discussed.^55,56^ Hence, some perturbation of ion fluxes in the electrolyte bulk occurs, whereby cations are adsorbed and counterions are repulsed from the electrode/electrolyte interface. Thus, a decrease in *S*_BET_ is more likely caused by both some additional redox reactions ongoing at the electrode/electrolyte interface (especially on the positive electrode) and by strong cation confinement/specific anion adsorption (or cation movement limitation in the negative electrode due to hydrogen sorption).^44^

It seems that the Li^+^ cation features do not follow the trends observed among other alkali metal cations (Na^+^, Rb^+^, Cs^+^). Figure 4 shows the exceptional features of lithium sulfate-based electrolytes. First, the capacitor with this electrolyte is assumed to have the shortest period of operation (due to the hypothesis of carbonate formation), which is not the case, as already discussed. Second, as it exhibits the best long-term performance, it can be supposed that the aging mechanism might be different. The smallest lithium dimension and the highest affinity for carbon π bonding allow the EDL to form effectively at the microporous carbon electrode/Li_2_SO_4_ electrolyte interface. However, textural changes of positive and negative electrodes subjected to a floating test with lithium sulfate do not distinguish them from others, suggesting that the aging mechanism is related to redox activity (the textural changes of positive electrodes are larger than those of negative ones). Additionally, because of the Li^+^ dimensions, electrode textural properties change to the smallest extent. Moreover, in the case of Li_2_SO_4_, the micropore volume of the negative electrode is not fully filled by Li^+^, leaving some volume available for efficient hydrogen sorption.^32^

When selecting a sulfate-based electrolyte for EC applications, one should consider the following various aspects depending on the application requirements:

(1) Energy output of the device: in this case, highly conductive rubidium or cesium sulfates are recommended.
(2) Price of the device: in this case, lithium sulfate is the most inexpensive electrolyte with the longest operation time.
(3) Long-term operation: lithium, rubidium, or cesium sulfate can be used as effective long-term operating electrolytes.

**Figure 5:**
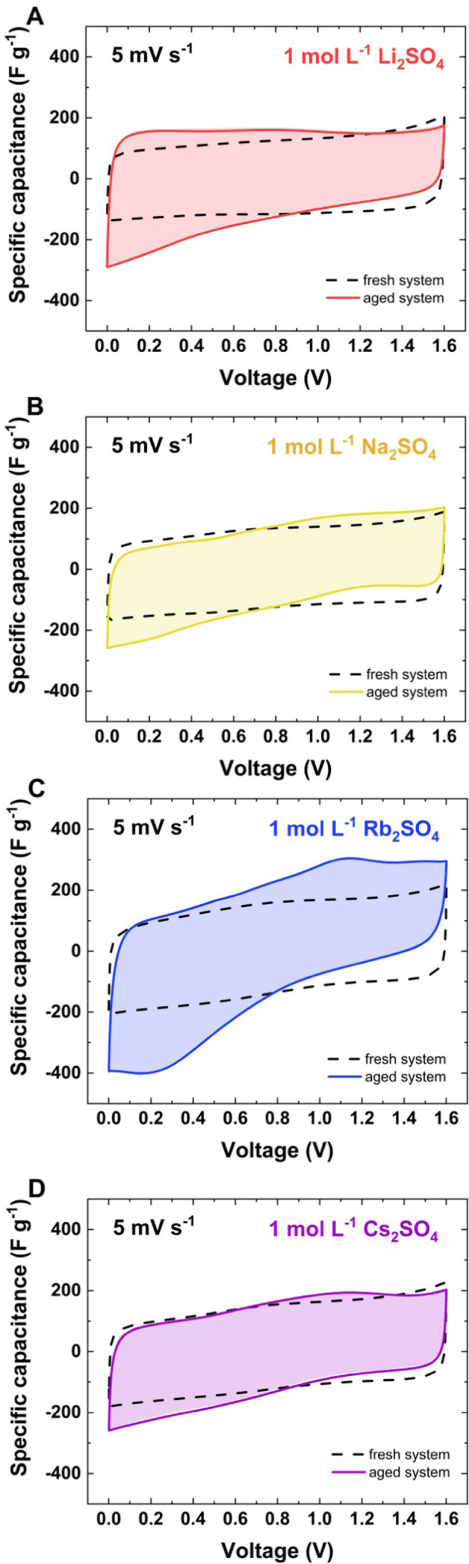
Cyclic voltammetry at 5 mV s^–1^ for fresh systems and systems after aging: (A) Li_2_SO_4_; (B) Na_2_SO_4_; (C) Rb_2_SO_4_; (D) Cs_2_SO_4_.

Some pseudocapacitive contribution is observed when comparing the cyclic voltammetry response recorded when the system is freshly built to that when the end-of-life criterion is met (*C*/*C*_0_ = 80%). Figure 5 shows the cyclic voltammograms measured at 5 mV s^–1^ for ECs with 1 mol L^–1^ M_2_SO_4_ (where M = Li^+^, Na^+^, Rb^+^, Cs^+^) for fresh and aged full systems. The three-electrode cyclic voltammograms presented in Figure S3c show a current increase recorded for the positive electrode, even though the ca. 10 mV s^–1^ scan rate applied is quite fast (as this experimental result resembles a galvanostatic charge/discharge profile at 1 A g^–1^). For the slower scan rate, this current peak is much more pronounced.

When the Li_2_SO_4_ solution is applied as the electrolyte, the CV shape does not change after the aging test (Figure 5A). Small deviations may be observed in the low voltage region, up to 0.5 V. Usually, such a CV curve shape can be correlated with hydrogen storage on a negative electrode.^57^ As discussed in ref (58), hydrogen sorption is more favorable in alkaline media than in acidic media. However, as described in ref (32), instead of the flux of sulfate anions, the movement of hydroxide species was detected; therefore, at the electrode/electrolyte interface, alkaline pH can be considered to favor the hydrogen sorption process rather than molecular hydrogen evolution. Therefore, sulfate-based electrolytes show hydrogen storage capability when combined with porous activated carbons.^59^ It has also been shown that after 10 h of constant polarization, electrosorbed hydrogen begins to recombine and form molecular hydrogen.^60^ Thus, a 2 h pulsed voltage held at 1.6 V does not necessarily lead to hydrogen evolution, but as discussed in the literature, hydrogen sorption can effectively occur.^32^ Taking into account all of this information, the CV shapes recorded after aging tests (Figure 5) are reasonable. For all studied sulfate-based electrolytes, the oxidation and reduction peaks are reversible. Interestingly, the highest pseudocapacitance contribution and the largest disproportion of stored charge are visible when 1 mol of L^–1^ Rb_2_SO_4_ (Figure 5c) is tested.

Nyquist plots are presented in Figure S5, showing a wide semicircle at high-frequency regions recorded for all aged ECs. This indicates that charge transfer reactions occur, and moreover, the time constant of these reactions is much lower than that of pure electrostatic attractions. Moreover, the shape of the Nyquist plot in the case of each aged sulfate-based system indicates that the charge storage mechanism changes from being controlled by EDL formation to being limited by ion diffusion. This proves that more oxygenated surface functional groups are created on the positive electrode, which is in accordance with other scientific publications.^29,30^ Furthermore, hydrogen binding to the carbon network on the negative electrode also limits pore accessibility for ionic species to create.^61^ For all sulfate-based ECs, an increase in the equivalent series resistance (ESR) and equivalent distributed resistance (EDR) can be observed. The positive and negative electrode resistances are higher than those of the fresh systems, and the resistance of diffuse layers on each electrode increases. The most interesting result is the increase in the radius of the semicircle, which can be directly correlated with the significant increase in resistance within porosity.^61^ The increase in charge transfer resistance can originate from the decrease in ionic species concentration and/or the presence of a reaction with electron transfer and/or deep ion confinement in the electrode pore structure (such as hydrogen stored at the negative electrode).^62^

Observations from both potentiodynamic (CV) and potentiostatic (EIS) experiments clearly indicate the same origin of the aging process. The negative electrode in sulfate-based neutral electrolytic solutions stores hydrogen (Figure S6), which over time is confined in the pore volume. In contrast, the positive electrode undergoes oxidation processes. All of the ongoing phenomena decrease the concentration of ionic species in the electrolyte bulk, simultaneously increasing the electrode resistance or pore accessibility by increasing the number of surface functionalities and creating a spatial obstacle for counterion adsorption during EDL formation at the electrode/electrolyte interface.

The increase in current observed in the cyclic voltammetry profile of the positive electrode (Figure S3) indicates the presence of a redox reaction at the electrode/electrolyte interface. Such redox activity of sulfate-based systems has already been reported when hydrogen sorption was investigated.^32,58^ Nevertheless, considering possible sulfate-based ionic species in aqueous solution present at the potential ca. +0.4 V vs NHE (Figure S2), it is difficult to identify the reaction responsible for the current increase. However, an *E*_0_ potential shift is observed after the floating test with 1 mol L^–1^ Li_2_SO_4_ and 1 mol L^–1^ Cs_2_SO_4_ (Figure S7). This may suggest that the electrolyte solutions become more acidic. The potential range of the positive electrode is observed to narrow during long-term operation (Figure S7). This phenomenon results from hydrogen sorption on the negative electrode (Figure S6), which widens its electrochemical potential window and enhances the high redox activity of the positive electrode. Figure S6 clearly shows that hydrogen sorption is more pronounced for the electrolyte with higher ionic conductivity (1 mol L^–1^ Cs_2_SO_4_). Hydrogen storage on the negative electrode in neutral sulfate-based electrolytes is evidenced by the well-pronounced hysteresis in the cyclic voltammograms when the potential is progressively decreased by −100 mV and the potential window of the negative electrode is increasingly cathodic (Figures S6 and S7). Moreover, a pronounced oxidation peak is also observed when the polarization is reversed (Figure S6). This peak is related to the oxidation of hydrogen confined in the negative electrode pore volume.^32,58^

In summary, sulfate-based systems undergo various redox reactions during EC operation. Such redox activity depends directly on the textural characteristics of carbon (more microporous material, higher redox activity; Figure S3), which indicates that micropores act as small chemical reactors. Among all examined sulfate-based electrolytic solutions (Li^+^, Na^+^, Rb^+^, and Cs^+^), lithium sulfate can be considered the best electrolyte candidate for aqueous ECs. ECs with lithium sulfate are characterized by the longest lifetime (extraordinary cyclability reported^32^ and the long floating test (214 h) up to *C*/*C*_0_ = 80%), satisfactory charge/discharge efficiency, and sufficient specific capacitance values (107 F g^–1^ at 1 A g^–1^ charge/discharge up to 1.6 V). Furthermore, Li_2_SO_4_ is the most inexpensive salt among alkali metal sulfate salts. The smallest change in the Nyquist plot and CV after the aging process for ECs with 1 mol L^–1^ Li_2_SO_4_ shows that the phenomena occurring are mostly reversible from an electrochemical point of view. For the next optimization steps, Li_2_SO_4_ is chosen as the main supporting electrolytic solution due to its superior performance compared to other electrolytes based on alkali metal sulfates.

Thus, to propose the optimization of sulfate-based ECs in terms of prolonging their lifetime, two approaches are applied and discussed separately based on the utilization of Li_2_SO_4_ electrolytic solution:

(1) Study of pH influence on EC aging in 1 mol L^–1^ Li_2_SO_4_.
(2) Bication (Li^+^ and Na^+^) sulfate electrolyte study.

### 3.2 pH Influence on 1 mol L^–1^ Li_2_SO_4_ Aging Process in ECs

This part of the investigation addresses the influence of pH on the aging process of ECs with 1 mol L^–1^ Li_2_SO_4_ electrolytic solution. The initial pH of the solution is 8.4. However, we have observed that batch-to-batch Li_2_SO_4_ purchased from the same supplier with the same purity (min. 99.8%) varies in terms of the pH of the aqueous solution from 3 to 9. Therefore, we found it crucial to determine the direct influence of pH on the EC lifetime. Three ECs with pH-adjusted electrolytes, i.e., 3, 7, and 11, were subjected to the floating protocol. To acidify the solution, H_2_SO_4_ was added, while for alkalization, LiOH was used. Thus, the cation (Li^+^) or anion (SO_4_^2–^) matched those already present in the electrolytic solution (Li_2_SO_4_). One cannot exclude the presence of H^+^ or OH^–^ in the initial aqueous solution, according to the water equilibrium state and pH-driven splitting reaction. Therefore, introducing sulfuric acid or lithium hydroxide into the electrochemical system does not change the solution composition qualitatively, and only the quantity of relevant ionic species varies in accordance with the pH studied. Furthermore, the prepared electrolytes are stable over time: their pH after three weeks of solution storage at ambient conditions varies less than 0.5%.

**Figure 6:**
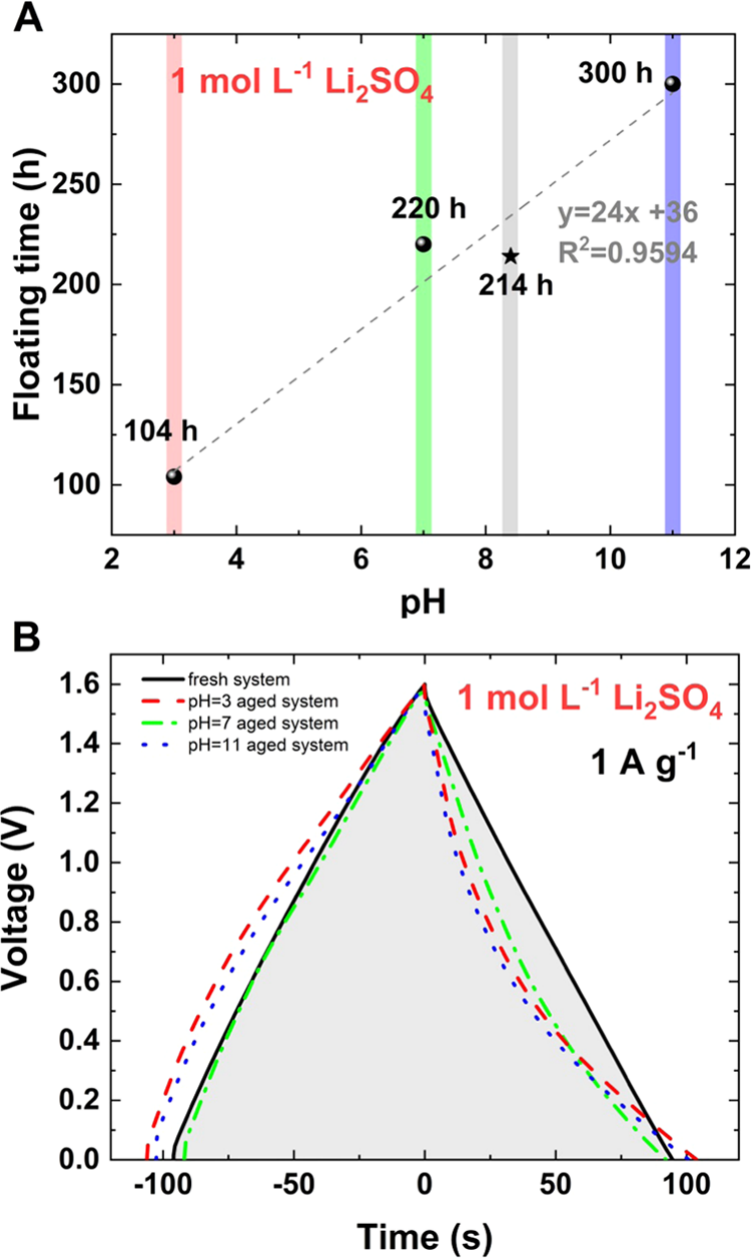
Study of the effect of pH on the EC aging process: (A) floating time vs pH of 1 mol L^–1^ Li_2_SO_4_ electrolytic solution. The system without pH adjustment (pH = 8.4) is denoted with a star symbol. (B) Constant-current charging/discharging at 1 A g^–1^ recorded for fresh systems and aged ones at different pH values.

The floating time vs pH of the electrolytic solution utilized in EC is shown in Figure 6A. A linear correlation is observed for the studied systems, with a high *R*^2^ factor (0.9594). The reference sample, therefore, without an adjusted pH (8.4) is denoted with a star symbol. In alkali media, effective electrosorption of hydrogen cannot be excluded, which has been previously reported for hydroxide- and sulfate-based solutions.^63,64^ Thus, our findings perfectly satisfy this assumption since a more acidic environment precludes hydrogen storage. Moreover, an initial alkaline environment favors hydrogen sorption on the negative electrode and enhances the specific capacitance evolution over time. The system utilizing 1 mol L^–1^ Li_2_SO_4_ at pH 3 operates for only 104 h of the floating test, while ECs with 1 mol L^–1^ Li_2_SO_4_ at close to neutral pH 7 exhibit 220 h (214 h for pH 8.4) of operation. The EC with 1 mol L^–1^ Li_2_SO_4_ at pH 11 operates for 300 h. Therefore, only a small adjustment of the pH strongly influences the operational time of the device (in the range ±50%) and determines which processes are favorable at the electrode/electrolyte interface. Furthermore, such findings indicate how crucial the electrolyte pH is for aqueous EC application, as it directly influences the stability of the system. Figure 6B presents constant-current charge/discharge curves recorded for fresh cells and aged cells with various electrolyte pH values. The charge/discharge curves recorded for fresh systems with electrolytic solutions of various pH values (pH 3–11) do not differ. Thus, the pH influences only the stability of the sulfate anion upon electrochemical operation. All systems were stopped at the same qualitative moment (*C*/*C*_0_ = 80%). Thus, the recorded curves do not differ very much from each other. However, at pH 3 and 11, the charging curve is observed to have a convex shape, indicating a deviation from the pure electrostatic charge storage mechanism. During the discharge process, the recorded curve trends are very similar with a slightly concave shape. The smallest qualitative change is observed when the pH of the electrolyte is adjusted to 7. Constant-current charge/discharge curves have been used to evaluate cell performance using an integral instead of the time of discharge.^65^ Considering coulombic characteristics, all systems might operate longer, as their discharge lasts a similar time. However, the real amount of energy delivered during discharge is smaller, and this is a crucial parameter for possible EC application.

**Figure 7:**
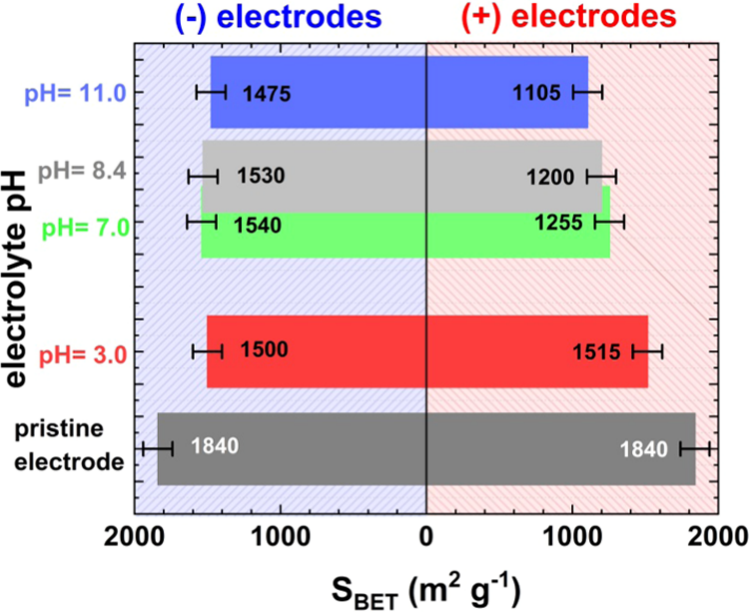
S_BET_ for (+) and (−) electrodes after the aging process of ECs with 1 mol L^–1^ Li_2_SO_4_ at various pH values.

The *S*_BET_ evolution of electrodes at various electrolyte pH values after the aging process is presented in Figure 7. The specific surface area (SSA) of negative electrodes decreases similarly, as the cation flux responsible for EDL formation is the same for all studied systems (Li^+^). The average *S*_BET_ observed for the negative electrodes is on the level of 1510 m^2^ g^–1^. Interestingly, the *S*_BET_ fade of positive electrodes varies remarkably when the pH of the electrolytic solution changes. It must be noted that the systems were stopped at the same *C*/*C*_0_ ratio of 80%. Hence, the operational time of each system is different. The evolution of *S*_BET_ over time (presented in Figure S8) shows that the decrease in SSA follows the operational time trend, as it is linear with a relatively high *R*^2^ coefficient (0.9732). When this is taken into account, the positive electrode *S*_BET_ fades as an effect of redox activity that directly influences the operating time of the device. Therefore, it is recommended to alkalize lithium sulfate solution prior to use to extend the EC lifetime.

### 3.3 Bication (Li^+^ and Na^+^) Sulfate Electrolyte in ECs

Two cations were introduced into an electrolyte solution containing SO_4_^2–^ anions. To date, only a single salt solution (either Li_2_SO_4_ or Na_2_SO_4_, etc.) has been tested as an electrolyte in ECs. Our interest is to verify whether the mixture of two sulfate-based electrolytes can improve the electrochemical properties of ECs in terms of their long-term performance. Li_2_SO_4_ solution was chosen as the main supporting electrolyte, and Na_2_SO_4_ was selected as the secondary electrolyte. Despite the fact that EC performance with 1 mol L^–1^ Na_2_SO_4_ does not differ much from that of the Li_2_SO_4_ system in terms of capacitance or textural changes after aging, unfortunately, it suffers from the shortest lifetime (114 h) during the floating test. Therefore, Na_2_SO_4_ solution was proposed as a second component of the bication electrolyte to prolong its lifetime and prevent Li_2_CO_3_ deposition.

The number of sulfate ions present in the bielectrolyte is twice as high as that in the single-cation 1 mol L^–1^ electrolytic solution (mixture 1). The second solution contains 0.5 mol L^–1^ Li^+^, 0.5 mol L^–1^ Na^+^ and 1 mol L^–1^ SO_4_^2–^ (mixture 2). The idea behind this is that in mixture 1, the number of Li^+^ and Na^+^ cations is the same as that in the one-cation solutions, i.e., 1 mol L^–1^ Li_2_SO_4_ or 1 mol L^–1^ Na_2_SO_4_. In mixture 2, the concentration of sulfate anions is the same as that in the one-cation solutions. This approach allows verification of whether the aging phenomena of sulfate-based ECs are related to the concentration of cations or SO_4_^2–^ anions.

**Figure 8:**
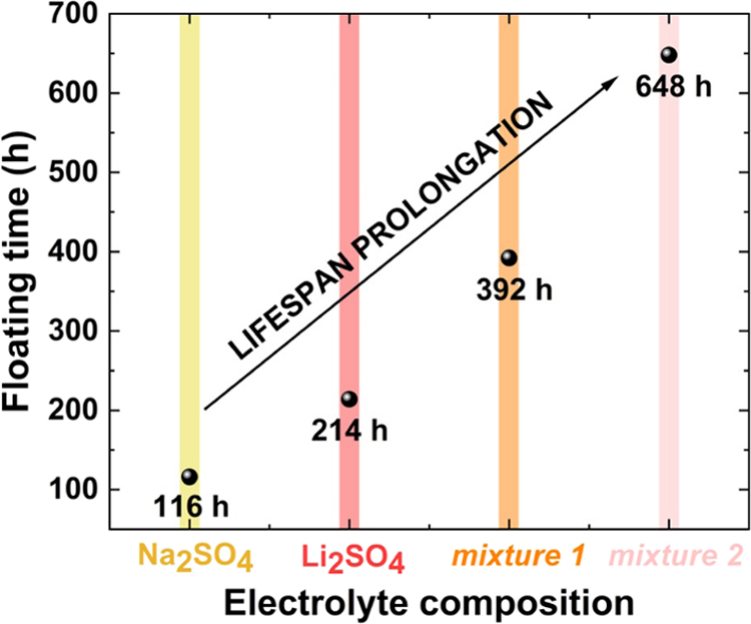
Floating time of ECs with sodium sulfate and lithium sulfate and their mixtures.

Table 3 summarizes data related to ECs with bication electrolytes, i.e., physicochemical characteristics of the electrolytic solution itself, floating time to reach the end-of-life criterion (presented also in Figure 8), *S*_BET_ values of positive and negative electrodes, and resistance changes calculated either from the ohmic drop during constant-current charging/discharging^61^ and impedance spectroscopy results at 0 V (ESR from EIS). Moreover, as aging of the full device is herein discussed, the change in EDR from impedance spectroscopy is also presented in Table 3 (EDR from EIS). In particular, for the single-cation electrolytic solution, the charge transfer region is more pronounced after the aging process (Figure S5), proving the presence of the redox contribution.

**Table 3:** Summary of Bication EC Long-Term Performance in Comparison with That of ECs with 1 mol L^–1^ Li_2_SO_4_ and Na_2_SO_4_

sulfate salt	concentration, mol L^–1^	σ, mS cm^–1^	floating time, h	*S*_BET_ (+) electrode, m^2^ g^–1^	*S*_BET_ (−) electrode, m^2^ g^–1^	resistance from GCPL, %	ESR from EIS, %	EDR from EIS, %
Li_2_SO_4_	1	72	214	1200	1530	+267	+128	+110
Na_2_SO_4_	1	83	116	1250	1540	+532	+143	+129
Li_2_SO_4_ + Na_2_SO_4_	1 + 1	102	392	975	1645	+631	+156	+153
	0.5 + 0.5	81	648	1190	1640	+288	+186	+158

Mixture 1 is characterized by a higher conductivity value (102 mS cm^–1^) related to more charge carriers present in the electrolyte volume, whereas mixture 2 displays a conductivity similar to that of 1 mol L^–1^ Na_2_SO_4_ (81 vs 83 mS cm^–1^). Therefore, from a physicochemical point of view, the mixtures do not stand out much from their one-cation constituents. Interestingly, from an electrochemical point of view, the lifetime of ECs with the mixed solution is prolonged to a great extent (+83% up to +200% compared to the floating time of ECs with 1 mol L^–1^ Li_2_SO_4_). First, what can be observed is that a more successful lifetime extension is achieved when mixture 2 is used as the electrolyte. Mixture 1, of which the concentration of SO_4_^2-^ anions is higher compared to that of mixture 2, meets the end-of-life criterion faster. Thus, this indicates that the aging of sulfate-based ECs is mostly connected with anion instability, proving also the influence of the electrolyte pH on the textural changes of the positive electrode, resulting from anion interactions (Section 3.2). Beneficial interactions of cations might improve the cycle life but definitely do not aggravate pore clogging, so the utilization of a bication electrolytic solution is an advantageous approach in EC applications.

In this context, we also compared the leakage current data during the EC aging process with individual electrolytes (1 mol L^–1^ Li_2_SO_4_ and 1 mol L^–1^ Na_2_SO_4_ and their mixtures; see Figure S9). The initial leakage current (before aging) of the capacitor with 1 mol L^–1^ Na_2_SO_4_ is very high (160 mA g^–1^), while that of the capacitor with 1 mol L^–1^ Li_2_SO_4_ is 13 times smaller (12 mA g^–1^). This is directly related to the mobility of Li^+^ ions and their better distribution in carbon pores. Interestingly, after meeting the end-of-life criteria, the leakage current value of the capacitor with 1 mol L^–1^ Li_2_SO_4_ increases to 60 mA g^–1^, while for the capacitor with 1 mol L^–1^ Na_2_SO_4_, the trend is the opposite, and a decrease in the leakage current decrease occurs (96 mA g^–1^). When carbonate is not formed, the leakage current generally decreases during the aging process. The capacitor with 1 mol L^–1^ Na_2_SO_4_ operates for a shorter time due to an unstable ion population at the interface, resulting from a relatively low cation binding energy to the aromatic carbon ring, as shown in Table 1. Indirectly, different kinds of interactions between the electrode and electrolytes are also observed in GC–MS studies, where more extensive gas formation is observed for the capacitor with 1 mol L^–1^ Na_2_SO_4_. Finally, the leakage current value of the capacitor with 1 mol L^–1^ Na_2_SO_4_ is always higher than that of the capacitor with 1 mol L^–1^ Li_2_SO_4_ as the electrolyte.

Both mixtures ensure a longer capacitor operating time due to the properties of cations. For both mixtures, one may observe a decrease in leakage current after aging, resulting from the denser ion packing within the carbon pores. However, the leakage current of the capacitor with mixture 2, the operation time of which is extremely long, is much lower than that of the capacitor with mixture 1. We postulate that this is most likely due to the perfect ion distribution and limited Li_2_CO_3_ formation.

Our findings are confirmed by the *S*_BET_ values of the electrodes after aging tests. The values of the negative electrodes, affected by cation adsorption/desorption, are comparable to the values obtained when unmixed electrolyte solutions (one-cation) are applied (1530 m^2^ g^–1^ for 1 mol L^–1^ Li_2_SO_4_, 1540 m^2^ g^–1^ for 1 mol L^–1^ Na_2_SO_4_ and ca. 1640 m^2^ g^–1^ for both mixtures). The *S*_BET_ of the positive electrodes is 1200 m^2^ g^–1^ for 1 mol L^–1^ Li_2_SO_4_ and 1250 m^2^ g^–1^ for 1 mol L^–1^ Na_2_SO_4_. When bication mixtures with the same concentration of sulfate anion (mixture 2) are used, a comparable *S*_BET_ of positive electrodes is observed, i.e., 1190 m^2^ g^–1^. Interestingly, when a higher concentration of sulfate anion is present in the electrolyte volume (mixture 1), more detrimental textural changes are observed after floating tests, and the *S*_BET_ value decreases to 975 m^2^ g^–1^. Moreover, the resistance values are worth discussing. First, the change in the resistance calculated from the ohmic drop is much higher than in the case of impedance studies. It is caused by different experimental conditions and the fact that the ohmic drop is affected by both the ESR and the EDR values.^66^ Moreover, the resistance recorded after repolarization in the high-voltage range, i.e., 1.6 V, represents more harsh conditions than those of the impedance studies at 0 V. Thus, the high-voltage region is more affected by aging phenomena, and it is recommended to be very precise with respect to the values reported and discussed during data analysis. Considering the impedance studies (at 0 V) presented in Table 3, the systems do not exceed the end-of-life criterion related to a 200% resistance increase, with respect to both the ESR and EDR values. However, the change in ESR from the constant-current charge/discharge curves of the ECs with mixture 1 (which contains more SO_4_^2–^ anions) is the highest among all studied systems and equals ca. +650%.

**Figure 9:**
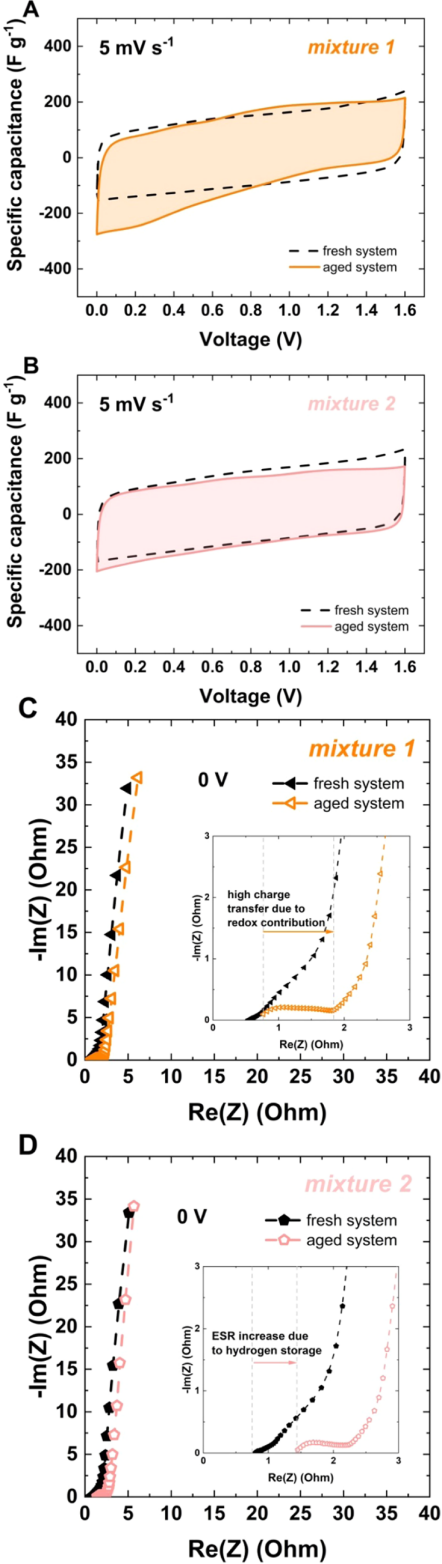
Cyclic voltammetry at 5 mV s^–1^ and Nyquist spectra at 0 V for a fresh system and after aging, with: (A, C) mixture 1 and (B, D) mixture 2.

Figure 9A,B presents cyclic voltammograms recorded before and after aging test at 5 mV s^–1^. It shows that EC with mixture 2, Figure 9B elicits an electrochemical response that changes negligibly from a qualitative point of view. This proves that this system is characterized by the most stable performance, and as a result, it can easily operate for a 648 h floating test, although the concentration of sulfate anions is the same as that used for the study of alkali metal sulfates (1 mol L^–1^). This value is competitive with that of other sulfate-based systems presented (ECs with 1 mol L^–1^ Li_2_SO_4_ at pH 11 operate up to 300 h). It also demonstrates that slight modifications of already known/proposed ECs are worth reconsideration. Electrolyte solutions still require careful research and detailed investigation. The Nyquist spectra presented in Figure 9C,D also prove the above-mentioned conclusions.

**Figure 10:**
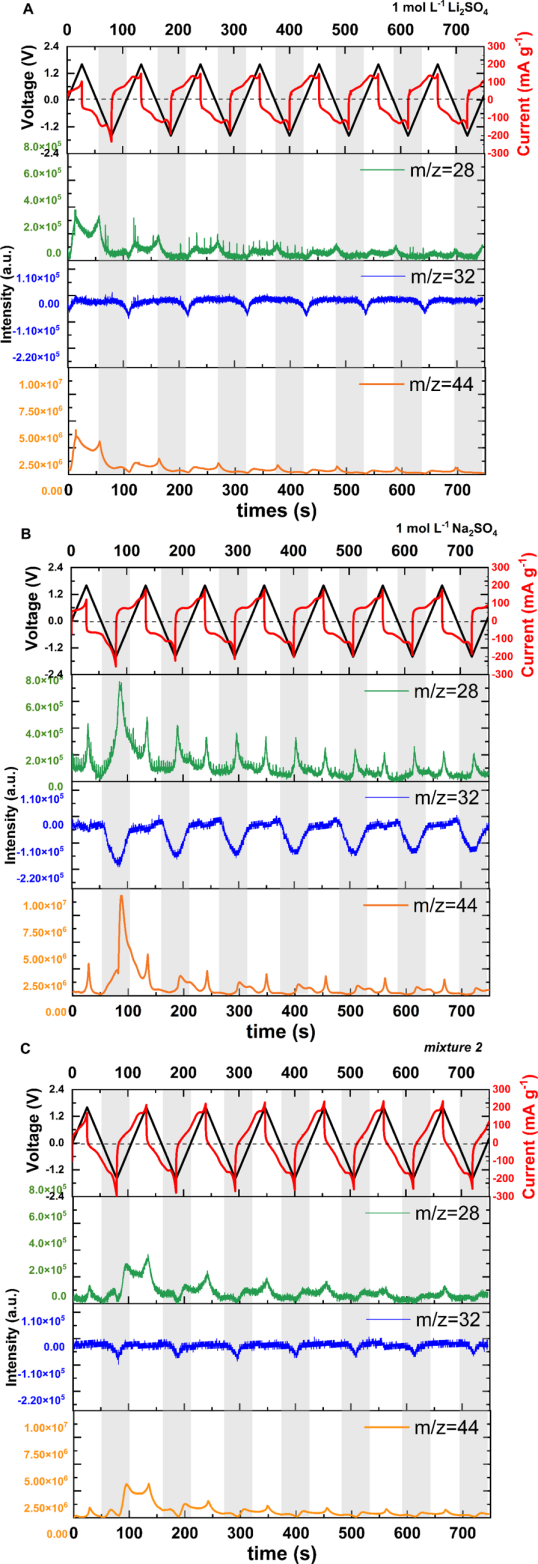
Profiles of selected mass spectra related to electrochemical response during 6 cycles of cyclic voltammetry at 1 mV s^–1^ for (A) 1 mol L^–1^ Li_2_SO_4_; (B) 1 mol L^–1^ Na_2_SO_4_; and (C) mixture 2.

Figure 10 shows the GC–MS spectra recorded in operando mode during EC operation.

Cyclic voltammetry was performed with a low scan rate of 1 mV s^–1^ to detect all individual changes and gases that evolved during the following cycles. Taking into account the results obtained during floating experiments, 1 mol L^–1^ Li_2_SO_4_ (Figures 10A), 1 mol L^–1^ Na_2_SO_4_ (Figure 10B), and their mixture (mixture 2: 0.5 mol L^–1^ Li_2_SO_4_ + 0.5 mol L^–1^ Na_2_SO_4_; Figure 10C) were selected for operando GC–MS experiments. Figure 10 shows various *m*/*z* ratios, which were selected as the representative ones. It is estimated that an *m*/*z* ratio equal to 28, 32, and 44 corresponds to CO, O_2_, and CO_2_, respectively. It was previously proven that during the operation of aqueous medium-based ECs at high voltages, there is CO_2_ and/or CO evolution.^33^ As a result, solid-state deposition occurs on the carbon electrode surface after long-term galvanostatic cycling or floating tests. On the basis of previous research, it is expected that mainly carbonates are formed during EC aging. Therefore, if lithium cations are used in electrolytes (e.g., LiNO_3_ or Li_2_SO_4_ as electrolytic solutions), it is estimated that lithium carbonate is deposited on the carbon surface, clogging the electrode pores and in effect lowering the specific capacitance over the operation time.^30,31^ As mentioned, Li_2_CO_3_ is poorly soluble in water. Therefore, Na_2_SO_4_ should be selected as a promising electrolyte for ECs to avoid salt precipitation. Nevertheless, as various aspects influence the overall capacitor performance, preventing deposit formation is not the only remedy for EC behavior improvement. The chosen electrolyte itself also has a significant impact. In effect, 1 mol L^–1^ Li_2_SO_4_ seems to be much better than 1 mol L^–1^ Na_2_SO_4_ even though solid-state deposit is eliminated in the latter case. An EC with 1 mol L^–1^ Li_2_SO_4_ operated for 214 h of floating, and an EC with 1 mol L^–1^ Na_2_SO_4_ operated for only 116 h. Therefore, to obtain a synergetic effect of both cations (Na^+^ and Li^+^), their mixture (assigned as mixture 2) was tested. A capacitance fade of 20% was obtained after 648 h for mixture 2, which indicates outstanding EC cycle life improvements. When *operando* mode GC–MS is considered, one may observe that for Li_2_SO_4_, Na_2_SO_4_, and mixture 2, there is an evolution of CO_2_ and CO during the following cycles. Slightly more CO_2_ and CO evolve in the case of Na_2_SO_4_ than for Li_2_SO_4_, which may explain the shorter floating time for Na_2_SO_4_ than for Li_2_SO_4_, as the internal gas pressure definitely increases and also increases the cell resistance. Interesting behavior may be observed when O_2_ (*m*/*z* = 32) is considered. In all cases, *m*/*z* = 32 decreases at the same time as CO and CO_2_ evolution increase. It is expected that there is O_2_ dissolution and the simultaneous evolution of CO_2_/CO during the cycles. This follows our assumption regarding deposit formation; since Na_2_CO_3_ is much more soluble than Li_2_CO_3_, there are completely different *m*/*z* = 32 curves for Li_2_SO_4_ and Na_2_SO_4_, suggesting that more carbonate is dissolved in the system with Na_2_SO_4_ solution. Taking into account the above-mentioned issues, one may observe that a synergetic effect is obtained for mixture 2, explaining its superior long-term performance (648 h of operation). A slightly smaller amount of CO_2_/CO is obtained due to the presence of Li_2_SO_4_, while the use of Na_2_SO_4_ eliminates the formation of solid-state deposits.

For both mixtures, the origin of the charge storage mechanism does not change: it still results from EDL formation even after the aging test. However, for one-cation sulfate electrolytes, the charge storage mechanism changes to the one controlled by ion diffusion (evidenced by the slope change in the Warburg region). Indeed, the pronounced electrolyte resistance semicircle (charge transfer resistance) increases significantly, but the same has been observed for all aged sulfate-based ECs. Such an increase in resistance with the preservation of satisfactory electrochemical performance may be explained by the high participation of hydrogen electrosorption. The H atoms are unlikely to evolve (after recombination), as their binding energy to the carbon matrix is relatively high (ca. 110 kJ mol^–1^).^67^

Mixture 2 is an inexpensive, stable, and durable electrolytic solution for EC application. It can be successfully applied in aqueous ECs, as its standard electrochemical and long-term performance is comparable to that of one-cation sulfate electrolytes for EC applications.

## 4 Conclusions

The electrochemical performance of ECs with sulfate-based electrolytes is discussed along with two successful approaches for extending their operational lifetime. The changes in the textural properties recorded for negative electrodes after the aging test correlate with cation type. However, in sulfate-based systems, the cation itself does not remarkably impact the long-term performance, as the aging mechanism is mostly related to the reversible oxidation of the positive electrode, balanced by hydrogen electrosorption on the negative electrode. Therefore, the alkalization of sulfate-based systems is recommended. The EC with 1 mol L^–1^ Li_2_SO_4_ with an adjusted pH of 11 operates longer (300 h) than the one with an acidic pH of 3 (100 h). Moreover, cation confinement in the micropore volume is observed. It is also important to consider the economic aspects of ECs with sulfate-based systems and the fact that Li_2_SO_4_ or Na_2_SO_4_ is the most inexpensive among alkali metal sulfate salts. Therefore, their bication mixture has been proposed to overcome the limitations of ECs with 1 mol L^–1^ Na_2_SO_4_. This approach is proven successful, as for two studied mixtures that vary in the concentration of their constituents, a significant lifetime improvement is observed, with up to 393 and 648 h of operation in the floating test. To date, they are the longest floating tests presented and discussed in the literature for water-based ECs. This also confirms our assumption that there is specific, ion-selective adsorption of ions, and the textural properties of the carbon electrode govern the mechanism of charge accumulation at the interface.

Our study demonstrates that the stability of sulfate-based ECs is strongly related to anion stability in terms of its concentration and pH. Redox-driven processes lead to shortening of the capacitor lifetime, especially if the SO_4_^2–^ concentration is higher than 1 mol L^–1^ and the pH is lower than 7. When an alkaline solution of sulfate salt is used, the EC lifetime is extended. However, the carbon positive electrode is more oxidized than in the acidic medium (evidenced by a high *S*_BET_ fade).

The utilization of bication sulfate electrolytes opens a new chapter for future improvements, and the variety of compositions should be thoroughly investigated.

## Supporting Information Available

Theoretical model for EDL formation in sulfate electrolytes; complementary activated carbon material (BP2000); specific capacitance vs floating time; Nyquist plots before and after floating; hydrogen sorption experiments for Li_2_SO_4_ and Cs_2_SO_4_; three-electrode galvanostatic profiles for Li_2_SO_4_ and Cs_2_SO_4_; S_BET_ of positive electrodes vs. operation time for Li_2_SO_4_; and leakage current recorded during aging for electrolyte mixture studies (PDF)
