# Supplementary material for: Ambiguous Role of Cations in the Long-Term Performance of Electrochemical Capacitors with Aqueous Electrolytes
Source: ACS Appl Mater Interfaces. 2023 May 4;15(19):23860–74. doi: 10.1021/acsami.2c21926 (PMC10197071; doi:10.1021/acsami.2c21926)
Supplement: Supplementary file 1 — am2c21926_si_001.pdf [file am2c21926_si_001.pdf]

Supporting Information

Ambiguous role of cations in long-term performance of electrochemical capacitors with aqueous electrolytes

Anetta Platek-Mielczarek†, Justyna Piwek, Elzbieta Frackowiak, Krzysztof Fic**

Poznan University of Technology, Institute of Chemistry and Technical Electrochemistry,

Berdychowo 4, 60-965 Poznan, Poland

†Laboratory for Multiphase Thermofluidics and Surface Nanoengineering, Department of Mechanical and Process Engineering, ETH Zurich, Sonneggstrasse 3, Zurich, Switzerland

*corresponding authors:

elzbieta.frackowiak@put.poznan.pl; krzysztof.fic@put.poznan.pl

List of content:

Table 1. Calculations of EDL formation for various sulfate-electrolytes in contact with polarized KYNOL 507-20 electrodes

BP2000 as a complementary microporous carbon used for electrochemical characterization:

Figure S1. Pore size distribution for microporous KYNOL 507-20 and micro- mesoporous BP2000 up to 50 nm

Figure S2. Specific capacitance *vs.* current density for two carbons KYNOL 507-20 (solid line) and BP2000 (dashed line) with 1 mol L^{-1} Li_2SO_4 and 1 mol L^{-1} Cs_2SO_4 electrolyte

Figure S3. Cyclic voltammetry profiles in the potential ranges determined from 1 A g^{-1} charge/discharge in 3-electrode set-up for Ecs with 1 mol L^{-1} Cs_2SO_4 A) BP2000 electrodes; B) KYNOL 507-20 electrodes

Figure S4. Relative specific capacitance evolution during floating test at 1.6 V

Figure S5. Nyquist plot for Ecs with M_2SO_4 electrolytic solutions at 0 V before and after ageing process: A) $M=Li^+$; B) $M=Na^+$; C) $M=Rb^+$; D) $M=Cs^+$

Figure S6. Hydrogen sorption in microporous KYNOL 507-20 carbon in: A) 1 mol L^{-1} Li_2SO_4; B) 1 mol L^{-1} Cs_2SO_4

Figure S7. Galvanostatic profiles at 1 A g^{-1} in 3-electrode set-up for fresh systems and aged ones (C/C0=80%) for Ecs with: A) 1 mol L^{-1} Li_2SO_4; B) 1 mol L^{-1} Cs_2SO_4

Figure S8. S_{BET} of positive electrodes vs. operation time of Ecs with 1 mol L^{-1} Li_2SO_4 with various pH

Figure S9. Leakage current data during the electrochemical capacitor ageing process with individual electrolytes (1 mol L^{-1} Li_2SO_4, 1 mol L^{-1} Na_2SO_4 and their mixtures

Table S1. Calculations of EDL formation for various sulfate-electrolytes in contact with polarized KYNOL 507-20 electrodes

Ionic specimen	Number of ionic species in 200 μL of electrolyte	Diameter of ionic specimen, cm	Volume of one ionic specimen (assuming ideal spherical shape), cm^3	Volume of all ionic species (of one type) in 200 μL of electrolyte, cm^3
Li^+	$1.20x10^{20}$	$4.20x10^{-8}$	$3.88x10^{-23}$	0.005
Na^+	$1.20x10^{20}$	$4.90x10^{-8}$	$6.16x10^{-23}$	0.007
Rb^+	$1.20x10^{20}$	$5.90x10^{-8}$	$1.07x10^{-22}$	0.013
Cs^+	$1.20x10^{20}$	$6.50x10^{-8}$	$1.44x10^{-22}$	0.017
SO_4^{2-}	$1.20x10^{20}$	$7.60x10^{-8}$	$2.30x10^{-22}$	0.028

Number of ionic species in 200 μL of electrolyte used in Swagelok® cell was calculated using Avogadro number (1 mol contains $6.022 x 10^{23}$ of ionic species). Carbon electrode of KYNOL 507-20 with an average mass of 9.5 mg contains 0.007 cm^3 of micropore volume (0.70 cm^3 g^{-1} from N_2 adsorption data). Therefore, all 1 mol L^{-1} sulfate electrolytic solutions provide enough ionic species to form uniform EDL at negative and positive electrode interface. Ionic species in electrochemical systems are supplied with excess considering formation of monolayer EDL. No other charge carriers are predicted to participate in EDL formation. Only for lithium cation there is still a room in micropore volume, that can be sufficiently used for hydrogen sorption.

In 1 mol L^{-1} Li_2SO_4 or Na_2SO_4, all cations can be stored in the micropore volume (total cation volume occupation for Li^+ and Na^+ is 0.005 cm^3 and 0.007 cm^3, respectively). Contrary, 1 mol L^{-1} solution of Rb_2SO_4 or Cs_2SO_4 provides an excess of charge carriers *vs.* the electrode micropore volume. Therefore, ca. 54% (for Rb_2SO_4) and 41% (for Cs_2SO_4) of cations forms EDL. The rest of cations are non-used and are still available in the electrolytic solution. It can be stated that EDL in all investigated sulfate-electrolytes can be formed uniformly at the negative electrode/electrolyte interface, i.e., a sufficient number of cations is available in a volume of

electrolyte used. In case of Li^+ there is still a room in pore volume that can be used for hydrogen sorption.

Considering adsorption of sulfate anion (with a solvated diameter of 0.76 nm [1, 2]), in each tested 1 mol L^{-1} electrolyte, only 25% of anions can be stored within the positive electrode's micropore volume and 75% remains in the electrolyte bulk. One can say, that big excess of SO_4^{2-} anions is present in the electrolytic solutions.

Of course, such scientific discussion assumes ideal spherical form of solvated ions and monolayer model of EDL that is not affected by other electrode features than its textural ones. However, discussed herein ions' dimensions and carbon pore size relationship is intended to emphasize that studied electrolytes provide excess of ionic species compared to the available surface area of electrodes in the cell. Thus, no limitations of EDL formation from electrolyte composition should occur.

BP2000 as a complementary microporous carbon used for electrochemical characterization

For comparison, micro-mesoporous carbon powder BP2000 was also used as electrode material with 1 mol L^{-1} Li_2SO_4 and 1 mol L^{-1} Cs_2SO_4 electrolytic solutions. BP2000 electrodes were prepared by mixing carbon in a powdered state (90 wt.%) with PTFE polymer binder (5 wt.%) and carbon soot (5 wt.%). Subsequently, ethanol used as a dispersive agent was evaporated at 80°C and electrode composite was then rolled into self-standing film. Electrodes with the same diameter as carbon cloth, (10 mm) were cut with an average mass of 6.0 mg (thickness ca. 150 μm) and used in the electrochemical cell.

BP2000 exhibits textural properties far different than self-standing carbon cloth (KYNOL 507-20). BP2000 consists of spherical carbon particles, which provide a developed external surface area, proven by a high mesopore ratio. On the contrary, carbon cloth is a typical microporous material.

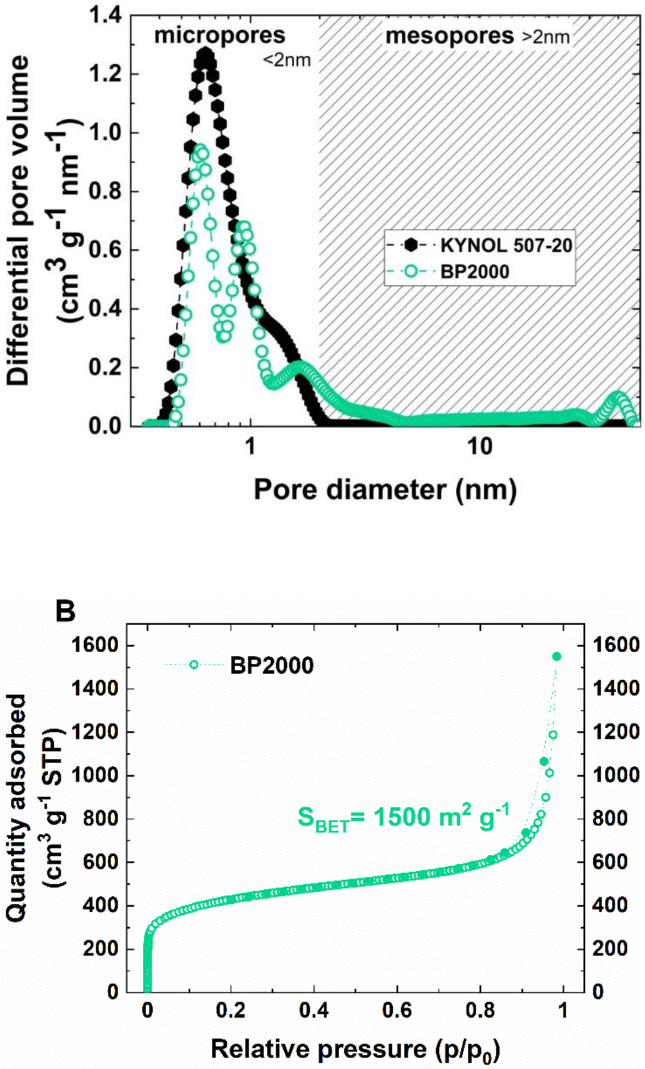

Figure S1. Textural characterization of carbon electrodes: A) pore size distribution for microporous KYNOL 507-20 and micro- mesoporous BP2000 up to 50 nm; B) adsorption isotherm with BET specific surface area value

BP2000 exhibits a high contribution of mesopores (V_{MESO}= 1.80 cm^3 g^{-1}) with micropore volume (0.50 cm^3 g^{-1}), with three L_0 values identified: L_{01}=0.65, L_{02}=0.95 and L_{03}=1.6 nm. Its adsorption

isotherm can be classified as type III. The high uptake of N_2 at high relative pressures is related to the external surface between carbon spheres in BP2000, visible in a pore volume peak at high pore diameter (Fig. S1A). BP2000 S_{BET} is 1500 $m^2\ g^{-1}$ (Fig. S1B) and S_{CUM} 1370 $m^2\ g^{-1}$.

Specific capacitance of Ecs with 1 mol L^{-1} Li_2SO_4 or Cs_2SO_4 electrolyte at different current loads and high voltage (1.6 V) is depicted in Fig. S2.

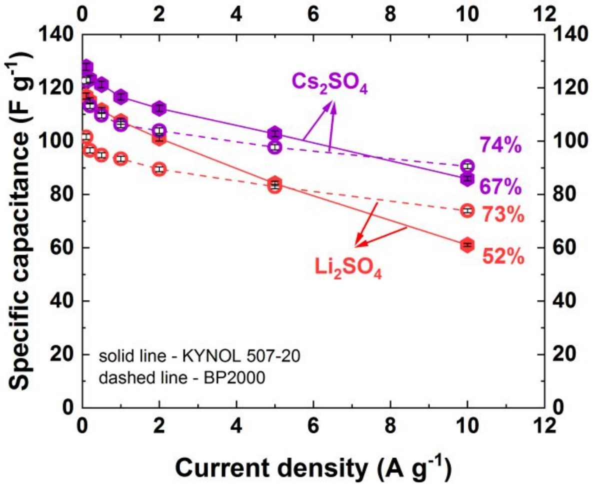

Figure S2. Specific capacitance *vs.* current density for two carbons KYNOL 507-20 (solid line) and BP2000 (dashed line) with 1 mol L^{-1} Li_2SO_4 and 1 mol L^{-1} Cs_2SO_4 electrolytes

One can notice that KYNOL 507-20 based Ecs with 1 mol L^{-1} Cs_2SO_4 exhibit a higher specific capacitance 86 F g^{-1} at 10 Ag^{-1}, while 61 F g^{-1} for Li_2SO_4. For a cesium-based system, not only

the specific capacitance is higher, but also the rate handling, i.e., 67% for Cs^+ *vs.* 52% for the Li^+-based system. Interestingly, when calculating the energetic efficiency of charge/discharge processes at high current loads, cesium-based Ecs demonstrate a more efficient charge storage process (73% at 10 A g^{-1}) compared to lithium-based one (60% at 10 A g^{-1}). However, at low current densities (0.1 A g^{-1}), the situation is opposite – energetic efficiency equals 70% for Cs_2SO_4-based system and 80% for Li_2SO_4-based one. Thus, for a relatively slow charge/discharge process, Li^+ seems to move more easily inside KYNOL 507-20 porosity than Cs^+. When the process is carried out at higher current loads (10 A g^{-1}), strong affinity of Li^+ for the carbon π bond decreases the efficiency of the process, as Li^+ tends to stay adsorbed at the electrode/electrolyte interface and the energetic efficiency drops to 60%. The opposite trend is observed for the Cs_2SO_4 solution. The higher mesopore volume in the BP2000 electrode results in a remarkable increase in the rate handling for both electrolytic solutions. Ecs with BP2000 electrodes and 1 mol L^{-1} Li_2SO_4 reveal 81% energetic efficiency at 10 A g^{-1} and with Cs_2SO_4 – 84%. This result suggests that the mesopore volume not only provides an open structure for organized ion movement but also ensures effective ion desorption during the discharge process [46]. A three-electrode study of the EC system with 1 mol L^{-1} Cs_2SO_4 and both carbons is presented

in Fig. S3. Calculated specific capacitance is slightly lower for BP2000 based systems. This difference can be explained by the less developed specific surface area of BP2000 (1500 m^2 g^{-1}) than the microporous KYNOL 507-20 (1840 m^2 g^{-1}).

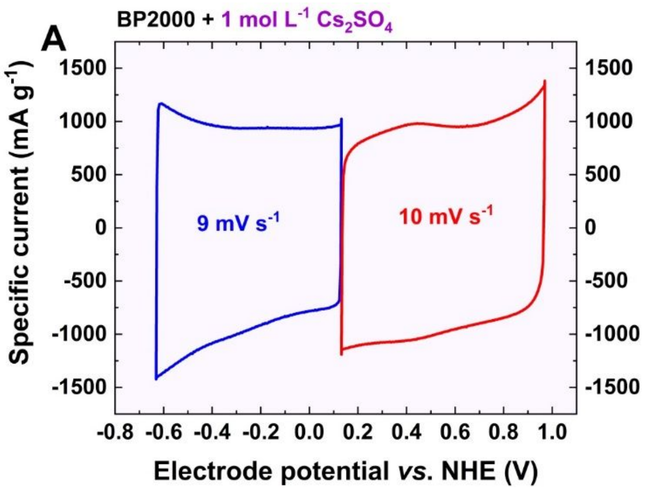

BP2000 + 1 mol L-1 Cs2SO4
A
Specific current (mA g-1)
9 mV s-1
10 mV s-1
Electrode potential vs. NHE (V)

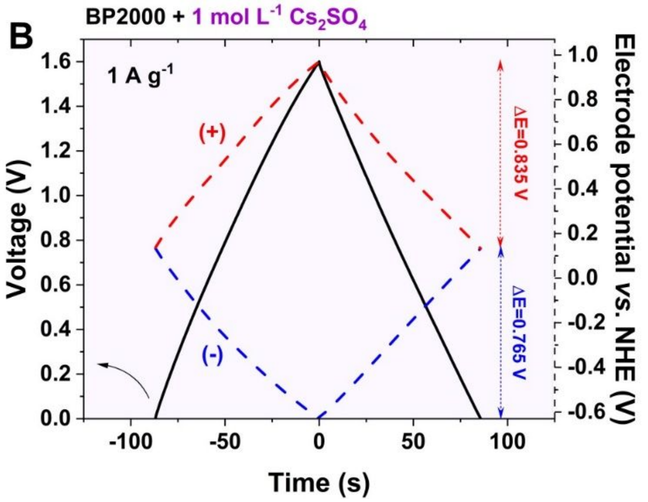

BP2000 + 1 mol L-1 Cs2SO4
B
1 A g-1
(+)
(-)
ΔE=0.835 V
ΔE=0.765 V
Voltage (V)
Electrode potential vs. NHE (V)
Time (s)

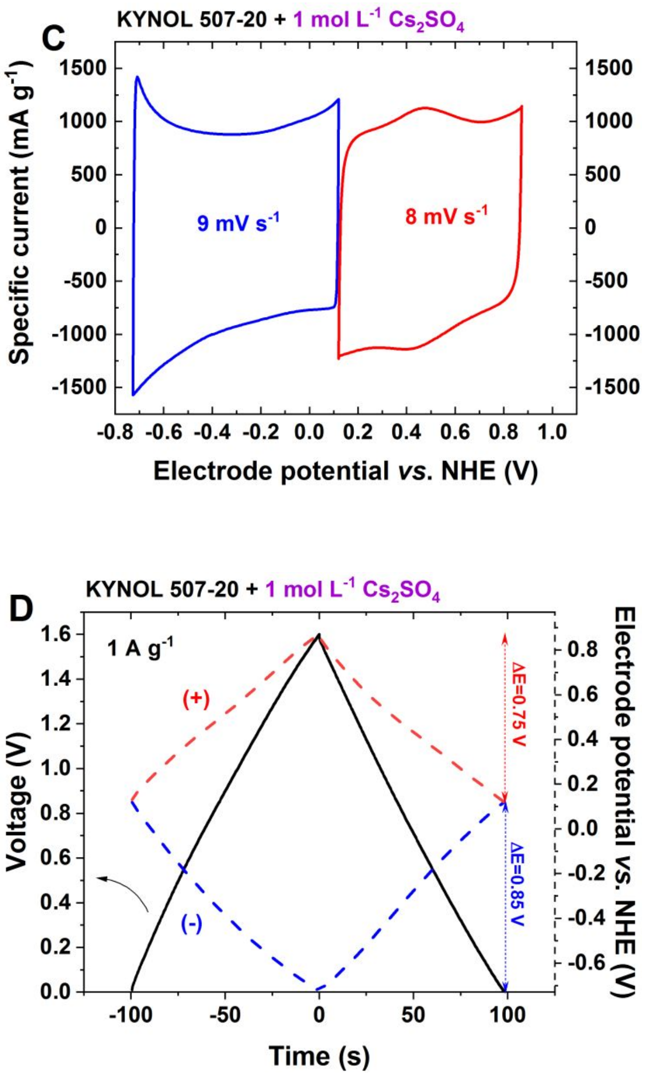

Figure S3. Cyclic voltammetry profiles in the potential ranges determined from 1 A g^{-1} charge/discharge in 3-electrode set-up for ECs with 1 mol L^{-1} Cs_2SO_4 A) BP2000 electrodes; B) KYNOL 507-20 electrodes

Ensuring higher mesopore volume, by applying BP2000 electrode material, one can observe increase in the rate handling for both electrolytic solutions (KYNOL *vs.* BP2000: 52% *vs.* 73% for Li_2SO_4 and 67% *vs.* 74% for Cs_2SO_4). Rate handling is an ability to store the charge at high current densities what directly influences power output of the device. Additionally, ECs with BP2000 electrodes and 1 mol L^{-1} Li_2SO_4 reveal 81% energetic efficiency at 10 A g^{-1} and with Cs_2SO_4 - 84%. This result suggests that mesopore volume not only provides textural open structure for organized ions movement, but also ensures effective ions desorption during discharge process. At low current densities, when mostly micropores take part in EDL formation, no big difference is observed between BP2000 and KYNOL 507-20 electrode materials.

Figure S3b and S3d presents full cell charge/discharge profiles and potential change over time for each electrode separately, i.e., positive (red) and negative (blue) recorded at 1 A g^{-1}. Although voltage of the cell is almost equally divided for two electrodes, two carbons with various textural properties exhibit different trends. When micro- mesoporus carbon is applied (BP2000), (+) electrode operates in a slightly wider potential window (0.835 V), whereas (-) in a narrower (0.765 V). Opposite situation is present for microporous KYNOL 507-20 electrode material. Cyclic voltammetry curves recorded after constant current charge/discharge at 1 A g^{-1}

with appropriate calculated scan rates for each electrode are presented in Fig. S3a and S3c. What can be observed in the positive electrode profile is the redox peak at potential ca. 0.4 *vs.* NHE, V. This current increase is more pronounced when KYNOL 507-20 is tested. In case of BP2000 smaller current peaks are observed at similar potential value. Even though charge storage mechanism in 2-electrode cell seems to be purely capacitive, small faradaic contribution cannot be neglected, especially when discussing positive electrode. One cannot omit that carbon oxidizes with time and surface functional groups may be the origin of observed peaks [3].

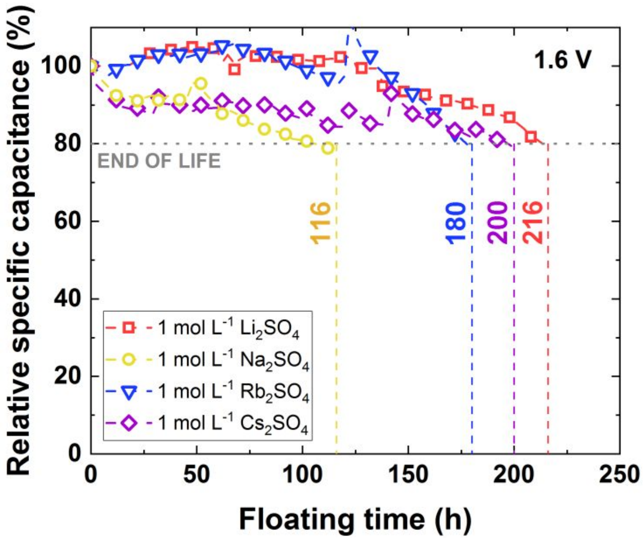

Figure S4. Relative specific capacitance evolution during floating test at 1.6 V

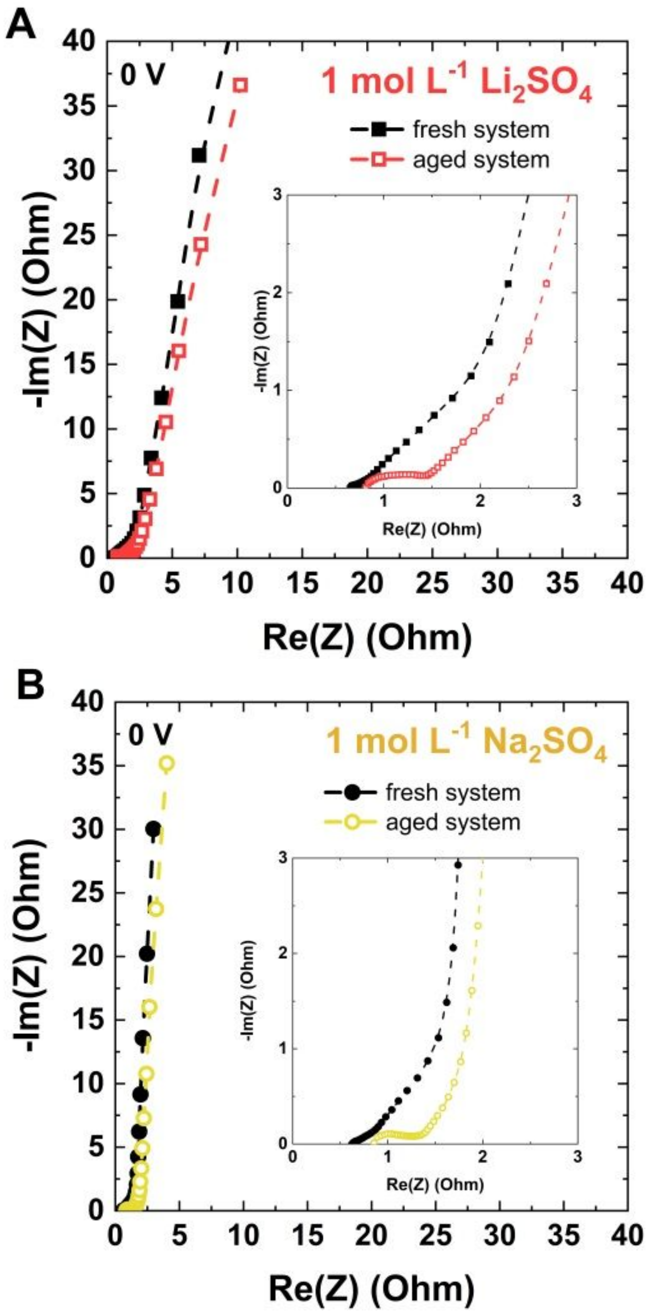
A
0 V
1 mol L-1 Li2SO4
fresh system
aged system
-Im(Z) (Ohm)
Re(Z) (Ohm)
B
0 V
1 mol L-1 Na2SO4
fresh system
aged system
-Im(Z) (Ohm)
Re(Z) (Ohm)

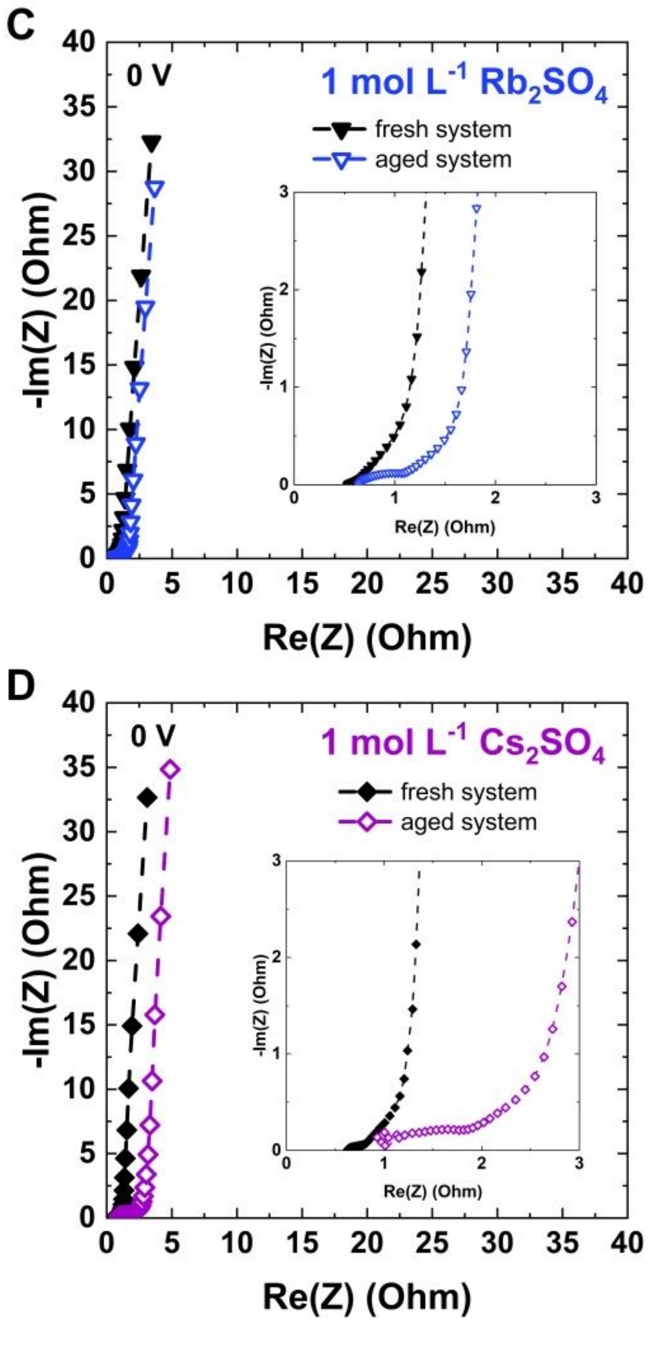

Figure S5. Nyquist plot for ECs with M_2SO_4 electrolytic solutions at 0 V before and after ageing process: A) M=Li^+; B) M=Na^+; C) M=Rb^+; D) M=Cs^+

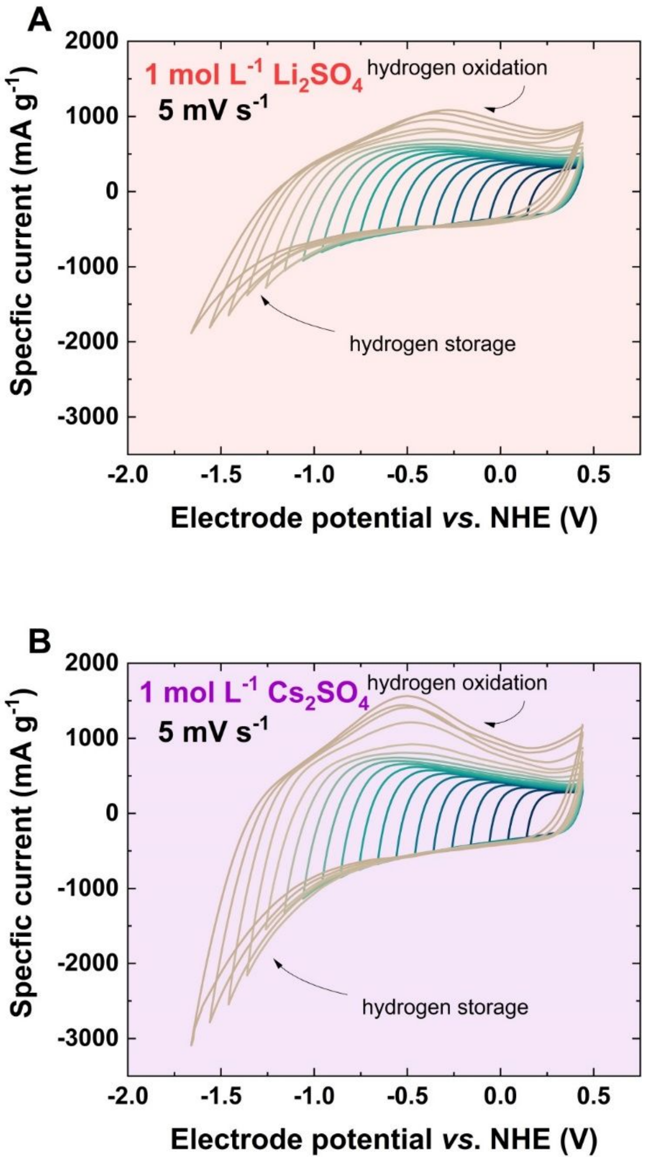

Figure S6. Hydrogen sorption in microporous KYNOL 507-20 carbon in: A) 1 mol L^{-1} Li_2SO_4;

B) 1 mol L^{-1} Cs_2SO_4

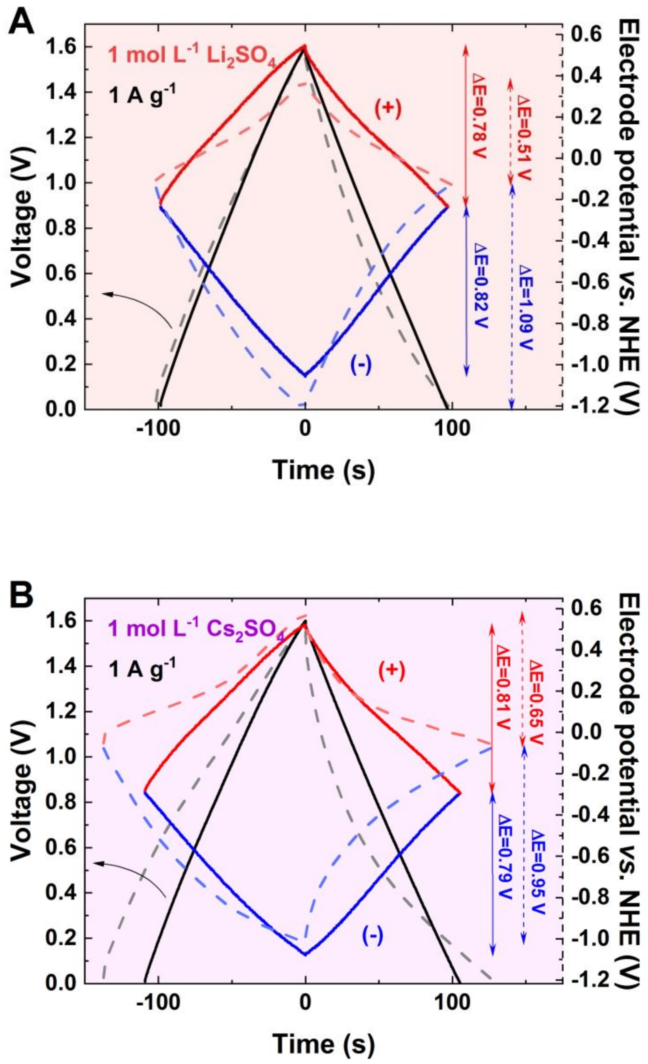

Figure S7. Galvanostatic profiles at 1 A g^{-1} in 3-electrode set-up for fresh systems and aged ones (C/C0=80%) for ECs with: A) 1 mol L^{-1} Li_2SO_4; B) 1 mol L^{-1} Cs_2SO_4

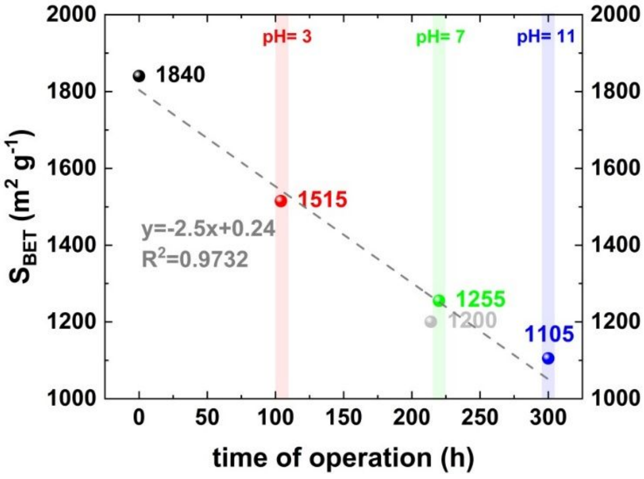

Figure S8. S_{BET} of positive electrodes vs. operation time of ECs with 1 mol L^{-1} Li_2SO_4 with various pH

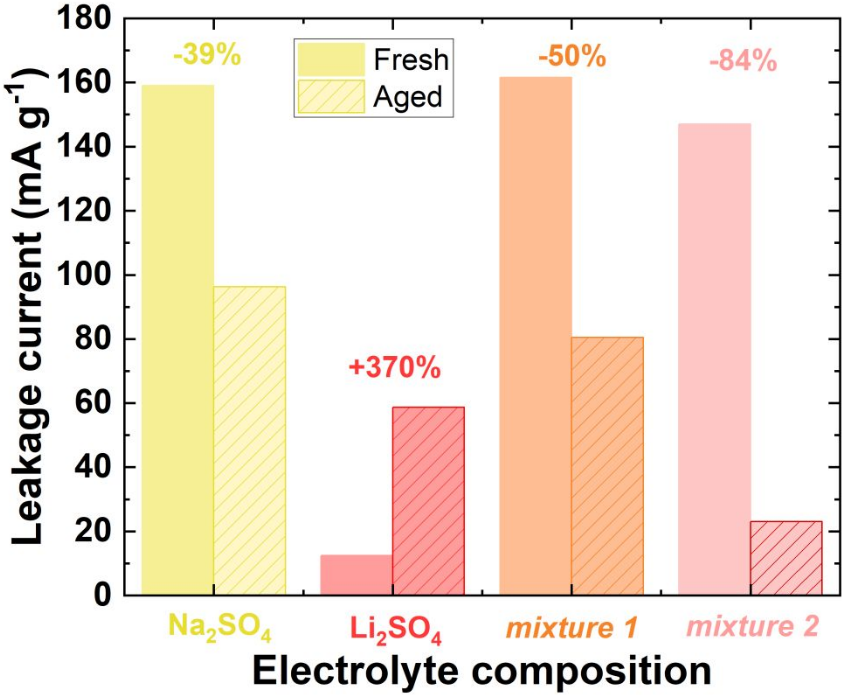

Figure S9. Leakage current data during the electrochemical capacitor ageing process with individual electrolytes (1 mol L^{-1} Li_2SO_4, 1 mol L^{-1} Na_2SO_4 and their mixtures

References

[1] Y. Chai, Z. Hu, W. Jia, H. Ji, Electrochimica acta, 356 (2020) 136780.

[2] Y.C. Wu, P.L. Taberna, P. Simon, Electrochemistry Communications, 93 (2018) 119-122.

[3] K. Fic, A. Platek, J. Piwek, J. Menzel, A. Slesinski, P. Bujewska, P. Galek, E. Frackowiak, Energy Storage Materials, 22 (2019) 1-14.
